# Identification of MicroRNAs and Target Genes in the Fruit and Shoot Tip of *Lycium chinense*: A Traditional Chinese Medicinal Plant

**DOI:** 10.1371/journal.pone.0116334

**Published:** 2015-01-14

**Authors:** A. B. M. Khaldun, Wenjun Huang, Sihong Liao, Haiyan Lv, Ying Wang

**Affiliations:** 1 Key Laboratory of Plant Germplasm Enhancement and Specialty Agriculture, Wuhan Botanical Garden, Chinese Academy of Sciences, Wuhan, Hubei, 430074, China; 2 University of the Chinese Academy of Sciences, Beijing, 100039, China; 3 Northwest Center for Agrobiotechnology (Ningxia), Chinese Academy of Sciences, Beijing, China; Nanjing Agricultural University, CHINA

## Abstract

Although *Lycium chinense* (goji berry) is an important traditional Chinese medicinal plant, little genome information is available for this plant, particularly at the small-RNA level. Recent findings indicate that the evolutionary role of miRNAs is very important for a better understanding of gene regulation in different plant species. To elucidate small RNAs and their potential target genes in fruit and shoot tissues, high-throughput RNA sequencing technology was used followed by qRT-PCR and RLM 5’-RACE experiments. A total of 60 conserved miRNAs belonging to 31 families and 30 putative novel miRNAs were identified. A total of 62 significantly differentially expressed miRNAs were identified, of which 15 (14 known and 1 novel) were shoot-specific, and 12 (7 known and 5 novel) were fruit-specific. Additionally, 28 differentially expressed miRNAs were recorded as up-regulated in fruit tissues. The predicted potential targets were involved in a wide range of metabolic and regulatory pathways. GO (Gene Ontology) enrichment analysis and the KEGG (Kyoto Encyclopedia of Genes and Genomes) database revealed that “metabolic pathways” is the most significant pathway with respect to the rich factor and gene numbers. Moreover, five miRNAs were related to fruit maturation, lycopene biosynthesis and signaling pathways, which might be important for the further study of fruit molecular biology. This study is the first, to detect known and novel miRNAs, and their potential targets, of *L. chinense*. The data and findings that are presented here might be a good source for the functional genomic study of medicinal plants and for understanding the links among diversified biological pathways.

## Introduction

*Lycium chinense* (Mill.), popularly known as Chinese boxthorn or goji berry and originating from China, is widely distributed in the temperate and subtropical regions of Japan, Korea and southeastern parts of Asia and is even found in European countries [1]. This species belongs to the nightshade (Solanaceae) family. The dry root bark and fruit of this plant have been used in traditional medicine, and young leaves have been widely consumed as a new functional vegetable. Numerous biochemicals, nutrients, minerals and vitamins in remarkable quantities have been reported [2]. The presence of protein, fat, carbohydrates, crude fiber, minerals, calcium, phosphorous, iron, carotene, thiamine, riboflavin, nicotinic acid and ascorbic acid in the dry fruits have ranked this species as one of the highest valued functional foods [1]. Although the mechanism is unknown, according to Chinese traditional medicine, this plant improves visual acuity [3]. The dry fruits of *Lycium* species also possess a wide range of clinical importance, such as anti-inflammatory effects [4], anti-aging qualities [5], anticancer and immunomodulating activities [6, 7], and blood glucose- and lipid level-reducing capabilities [8]. Although this species has many fascinating aspects, there is limited genome and molecular information. The gene regulation and functions of miRNAs need to be elucidated in this species.

A group of RNAs with a low molecular weight, termed small RNAs (sRNAs), possess tremendous regulatory functions. Small RNAs are classified into the following two categories depending on their biogenesis and action: short-interfering RNAs (siRNAs) and microRNAs (miRNAs) [9, 10]. Among these sRNAs, miRNAs are non-coding entities that are endogenous, 20–24 nt long and widely found in eukaryotes [11, 12]. These miRNAs regulate gene expression at the transcriptional and post-transcriptional levels [13], and play a crucial role in plant growth and development [10, 14, 15]. MicroRNAs are processed from the stem-loop position of long primary transcripts by a Dicer-like (DCL) enzyme in plants, and loaded into silencing complexes, where they cleave the complementary sequence of targets [16]. Plant miRNAs have only been known for approximately a decade [17], but are already known to play pivotal roles at every major developmental stage, especially in core gene regulatory networks, and are also known to regulate transcription factors (TF). Therefore, miRNAs are considered as “master regulators” [12, 18].

In fact, the large number of miRNAs that are evolutionarily conserved from mosses and ferns to higher flowering plants have been used as helpful indicators for the prediction of miRNAs using homology searches across various species [19, 20]. Recently, next-generation high-throughput sequencing technologies have permitted the efficient identification and quantification of miRNAs with high precision, which dramatically facilitates the understanding of miRNAs. Moreover, miRBase (The miRBase database is a searchable database of published miRNA sequences and annotation, http://mirbase.org/) has released a new version (v20, June 2013) with 24,521 miRNA loci, leading to the production of 30,424 mature miRNAs products belonging to 206 species [21]. Among the plant species, the majority of miRNAs have been identified in the monocot *Oryza sativa* and the eudicots *Populus trichocarpa* and *Arabidopsis thaliana*. After deep sequencing, miR167, miR1857 and miR172a were identified in sweet orange and have regulatory effects in lycopene accumulation [22]. There are also reports elucidating the molecular biology of different miRNAs related to fruit ripening, softening, ethylene biosynthesis and the signal transduction of different phytohormones [23–26].

Although miRNAs have been thoroughly studied in many species, no miRNAs have been identified so far from goji, and none are listed in the miRBase. Many studies have confirmed that both species-specific and conserved miRNAs are very important for plants in different biological processes. Therefore, profiling miRNAs in a non-model plant species is very important for understanding the evolutionary trend of miRNAs and the regulation of different biological processes. In this study, high-throughput sequencing was performed for the RNAs of young shoot tips with adjacent leaves and the bulk mixing of three developmental stages of fruits of *L. chinense*. We depict an overall scenario of miRNAs of the vegetative tissues (shoot and leaf) and reproductive tissues (three developmental stages of fruit) of this species, and then target genes that are related to fruit development as identified through significant differentially expressed miRNAs. Comparative studies and expression differences have revealed an interesting pattern and distribution of miRNAs between vegetative (shoot and leaves) and reproductive (fruit) tissues. Therefore, the miRNAs and targeted genes that were identified in this study are unique resources for biological pathway analysis and functional characterization. These data could be an exclusive source of comprehensive understanding of miRNA expression profiling in *L. chinense*, as it is an important representative of medicinal plants.

## Materials and Methods

### Plant materials

Healthy and non-lignified shoot tips (approximately 1 cm from the tip end), including adjacent leaves of *L. chinense* (cv. Large leaf goji), were collected from two-month-old plants that were grown at the Wuhan Botanical Garden of Chinese Academy of Sciences (CAS), Wuhan, China. To collect the optimum stages of fruits, three developmental stages were chosen, green, color breaking and red mature (S2 Fig). The shoot and leaves were mixed together for total RNA extraction, but individual stages of fruit were extracted separately followed by equal bulk mixing for sequencing and subsequent qRT-PCR and RLM 5’-RACE experiments. The tissues collected in sealer bags and snap-frozen immediately in liquid nitrogen. The target tissues were stored in a -70°C ultra-refrigerator for later and/or further uses.

### Total RNA isolation, purity check, sRNA library construction, and Illumina sequencing

The total RNAs were isolated from the tissue samples via the Trizol method with a slight modification following the manufacturer’s instructions of the RNAiso Plus (Takara) extraction kit. RNA degradation and contamination were monitored on 1% agarose gels, and purity was checked using the Nano photometer (Implen, CA, USA). The RNA concentration was measured with a Qubit 2.0 Fluorometer (Life Technologies, CA, USA), and the integrity was assessed by Agilent bioanalyzer 2100 (Agilent Technologies, CA, USA). The purified RNAs were used to construct small RNA libraries and were sequenced on an Illumina HiSeq 2500/2000 platform at the Novogene Company, Beijing, China.

### Bioinformatics analyses of sequencing data

Raw sequence data were obtained via the RNA high-throughput sequencing process. Raw reads were screened out to remove the contaminating reads, sequences containing ‘adapters’, without insert tags, and reads with poly-A tails. Sequences from 18–40 nt in length were used for further analysis. Clean reads of sRNA tags were mapped to the *L. barbarum* transcriptome database with less than 2 bp mismatch (provided by South China Botanical Garden, CAS, Guangzhou, China) using Bowtie [60]. Tags matching non-coding RNAs (rRNAs, tRNAs, snRNAs, and snoRNAs) were BLAST-searched with the sequences of Rfam (Rfam: http://www.sanger.ac.uk/software/Rfam) and the NCBI GenBank databases (GenBank: http://www.ncbi.nlm.nih.gov/blast/Blast.cgi) and deducted for downstream analyses [61].

The remaining sRNA tags were used to search the conserved or known miRNAs in miRBase 20.0 allowing a maximum of two mismatches [62]. The modified software miRDeep2 [63] and srna-tools-cli were used to obtain the potential miRNA and draw the secondary structures. The characteristics regarding the hairpin structure of the miRNA precursor were used to predict the potential novel miRNAs. With the integrated use of the software miREvo [64] and miRDeep2, we predicted the potentially novel miRNAs by exploring the secondary structures, the DL1 cleavage sites and the minimum free energy (MFE) of the sRNA tags that were unannotated in the former steps.

To explore the occurrence of miRNA families, we used the known miRNAs in miFam.dat (http://www.mirbase.org/ftp.shtml), and the novel miRNA precursors were aligned to Rfam (http://www.rfam.sanger.ac.uk/search/). To predict the target genes of miRNAs, psRobot.tar was used in psRobot software [65].

After predicting the target genes, the expression levels of miRNAs (quantification of miRNA) were estimated using TPM (transcripts per million) according to the normalization formula that was described by Zhou et al., 2010 [66]. The DEGseq R package (to minimize the positive false discovery rate for the sequencing data without biological replicates) was used (2010) to identify differentially expressed miRNAs between the two samples. The corresponding *P*-values were adjusted to a q-value [67]. The threshold level of the *P*-value was set as *P*-value<0.01 and ׀log_2_(foldchange)׀>1 for the significant differential expression.

The dataset that was studied in this article is available in the NCBI (SRA) public repository under the accession number SRP043345.

### Validation of miRNA expression using qRT-PCR

Poly (A)-tailed qRT-PCR was used to measure the gene expression variation and validate the deep sequencing results of miRNAs. The reverse-transcription reaction and real-time PCR primer design were conducted according to Rui Shi [68] and the manufacturer’s instructions (Takara-Prime script miRNA qPCR starter kit, 2.0), where each PCR reaction was performed in a volume of 25 µl containing 12.5 µl of SYBR premix ex Taq II (2×), 1 µl of each forward primer, 1 of µl universal reverse primer and reverse-transcribed cDNA from ~100 pg of total RNA. The PCR protocol was 5 seconds at 95°C, 40 cycles of 95°C for 5 seconds, 60°C for 20 seconds and 72°C for 1 minute. The primers that were used in this study are listed in Additional file 7. Real- time PCR was performed on the Applied Biosystem 7500 detection system using the SYBR Green I method, and all of the reactions were run in biological triplicates. The melting curve was used to determine the specificity of PCR products (primer amplicons).

To validate the reliability of Illumina throughput sequencing technology, 14 miRNAs were selected, 7 of which were randomly selected from known miRNAs and 7 from novel miRNAs after determining the appropriate reference genes. The delta-C_T_ (corresponding cycle threshold) method was used to calculate the relative expressional levels of miRNAs [69]. In this method, the C_T_ values of the desired miRNAs and reference genes were first transformed for measurement using delta-C_T_ followed by dividing the quantities of desired miRNAs by the geometric mean of the reference genes. The standard deviation and mean were calculated using triplicate qRT-PCR assays. The C_T_ was calculated using the machine accessory software and converted into relative copy numbers using a standard curve as previously described [30]. The gene 5.8 S rRNA was used as reference gene in the qPCR detection of miRNAs. Student’s t-test was used for the statistical analysis of the qRT-PCR.

### Functional assignments for potential target genes

There are three distinct segment of GO: biological process (BP), cellular component (CC) and molecular function (MF). For the GO enrichment analysis, the over-represented *P*-value and the corrected *P*-value identified the highly significant gene categories in the GO enrichment analysis followed by KEGG database searching to determine the functional diversity of the ‘target gene candidates’ of differentially expressed miRNAs [70]. Moreover, KOBAS [71] software was used to test the ‘target gene candidates’ in the KEGG pathways.

### Validation of predicted targets using RLM 5’-RACE

For the validation of candidate targets, modified RNA Ligase-Mediated 5’-RACE (RLM 5’-RACE) was performed. 5’-Full RACE Kit (Takara) was used according to the manufacturer’s instructions, with slight modifications. Briefly, total RNA was directly ligated to the 5’ adaptor followed by reverse transcription with the oligo (dT) primers. PCR was performed with 5’ primers and 3’ gene-specific primers using the cDNA as the template (S7 Table). The 5’-RACE PCR products were purified using Takara PCR product recovery kit, cloned, and sequenced.

## Results

### Summary of small-RNA library dataset by deep sequencing in *L. chinense*

**Figure 1:**
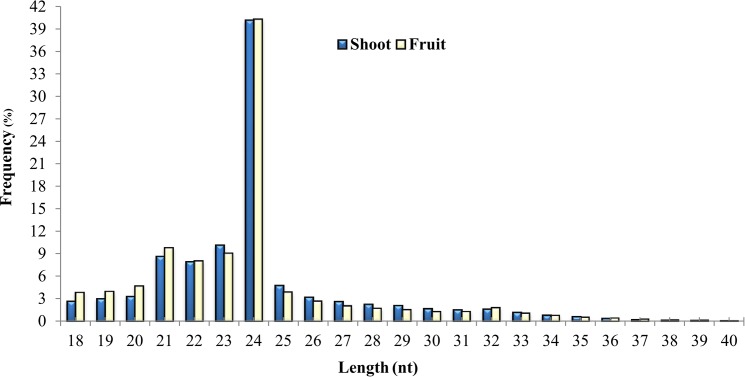
Length distribution of small RNAs in the shoot and fruit libraries of *L. chinense*. X-axis, length of sRNA distribution; Y-axis, corresponding percentage of raw reads.

To explore the regulatory networks of miRNAs in the fruit and shoot of *L. chinense*, we constructed two libraries from the young shoot tip with leaves and fruit tissues. To identify the target genes and regulatory pathways, Illumina sequencing technology was used. A total of 19,014,652 raw reads were obtained, of which 9,128,886 and 9,885,766 raw reads were produced in shoot and fruit sRNA libraries, respectively (Table 1). Moreover, 97.27% and 97.62% raw reads of high quality for the shoot and fruit libraries, respectively. For further study, 18 to 40 nt total small RNA sequences were selected (Fig. 1), with 21–24 nt long sequences with high frequency. The frequency of shoot sRNA was 40.19% (24 nt), followed by 10.14% (23 nt), 8.63% (21 nt) and 7.92% (22 nt), while this frequency was 40.33% (24 nt) followed by 9.83% (21 nt), 9.11% (23 nt) and 8.09% (22 nt) in the fruit sRNA library. The percentage of small RNAs of 24 nt was significantly higher than that of other sRNAs.

**Table 1:** Statistical summary of the data that were generated by high-throughput small-RNA sequencing in goji.

**Read type**	**sRNA libraries**		**Total**
	**Shoot**	**Fruit**	
Total raw reads	9,128,886 (100%)	9,885,766 (100%)	19,014,652
High quality	8,879,712 (97.27%)	9,650,746 (97.62%)	18,530,458
Low quality reads	3007 (0.03%)	6160 (0.06%)	9167
N%> 10%	3612 (0.04%)	0	3612
5’ adapter contaminants	3124 (0.03%)	6104 (0.06%)	9228
3’ adapter null	187,402 (2.05%)	189,023 (1.91%)	376,425
Poly (A/T/G/C)	52,029 (0.57%)	33,733 (0.34%)	85,762
Total bases (G)	0.456	0.494	0.95

N%, undetectable gaps at base calling.

Based on a trans-species alignment, the small RNAs were mapped against the Goji berry (*Lycium barbarum* L.) transcriptome database as provided by the South China Botanical Garden (SCBG, CAS, Guangzhou, China). A total of 3,826,551 (44%) and 3,278,589 (43.94%) unique small RNA reads were mapped for the shoot and fruit libraries, respectively. These mapped small RNAs comprised known miRNAs, putative novel miRNAs, rRNAs, tRNAs, snRNAs, snoRNAs, repeat-associated RNAs, TAS (trans-acting small interfering RNAs) and unannotated fragments (Table 2). Additionally, unannotated reads accounted for 81.70% and 82.76% in the shoot and fruit libraries, respectively.

**Table 2:** Number of reads for each small RNA classification as identified in *goji*.

**Read type**	**sRNA libraries**		**Total**
	**Shoot**	**Fruit**	
Total	3,826,551 (100%)	3,278,589 (100%)	7,105,140
Known miRNAs	22,966 (0.60%)	31,058 (0.95%)	54,024
Novel miRNAs	12,519 (0.33%)	28,187 (0.86%)	40,706
rRNAs	566,742 (14.81%)	425,905 (12.99%)	992,647
tRNAs	20,599 (0.54%)	11,522 (0.35%)	32,121
snRNAs	2886 (0.08%)	1715 (0.05%)	4601
snoRNAs	11,651 (0.30%)	3832 (0.12%)	15,483
Repeat associated RNAs	51,953 (1.36%)	52,636 (1.61%)	104,589
TAS	10,907 (0.29%)	10,243 (0.31%)	21,150
Unannotated	3,126,328 (81.70%)	2,713,491 (82.76%)	5,839,819

Known miRNAs, perfect matching mature miRNA sequences in the miRBase miRNA repository; Novel miRNAs, new mature miRNA sequences that were detected by characteristic stem-loop hairpins and minimum free energy (MFE); rRNAs, tRNAs, snRNAs, and snoRNAs are non-coding sRNAs; TAS, transacting small interfering RNAs; Unannotated, reads that had no known information.

### Identification of conserved and evolutionarily known miRNAs

To identify the known miRNAs, the sRNA libraries were BLASTN-searched for known mature plant miRNAs that were deposited in miRBase 20.0 (v20, June 2013). Following BLASTN search and subsequent sequence analyses, 94 known hairpin structures were identified corresponding to 60 unique mature miRNA sequences that were orthologs of conserved miRNAs from other plant species in the shoot and fruit libraries of *L. chinense*, representing 31 diversified miRNA families (S1 Table). In addition, among these 60 known miRNAs, 53 and 46 miRNAs were expressed in the shoot and fruit libraries, respectively.

**Figure 2:**
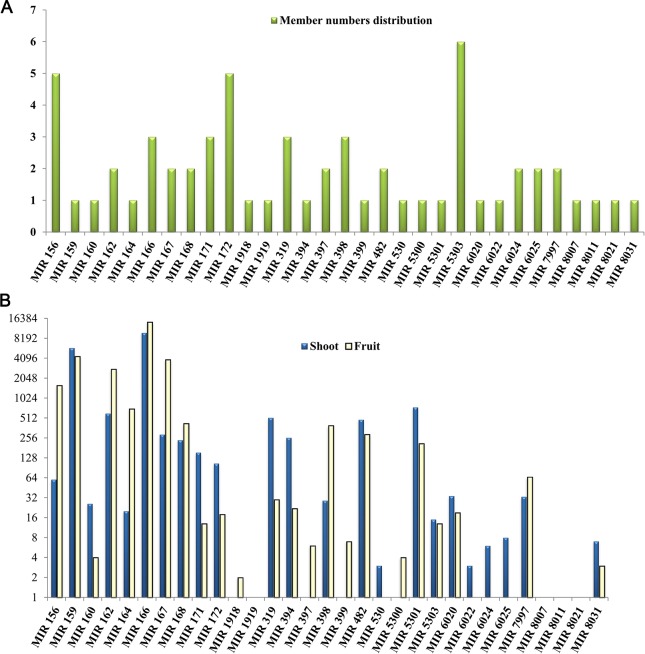
The distribution of the member numbers (A) and read counts of each known miRNA families (B) in *L. chinense*. X-axis, name of the miRNAs family; Y-axis, representing miRNA numbers.

The member numbers of different miRNA families were also analyzed (Fig. 2), which showed that approximately half of the families contained more than one member, while the other half had a single member. The families, MIR162, MIR166, MIR167, MIR168, MIR171, MIR319, MIR397, MIR398, MIR482, MIR6024, MIR6025 and MIR7997 possessed 2–3 members, while MIR156, MIR172 and MIR5303 contained 5, 5 and 6 members, respectively. Sixteen families, namely MIR159, MIR160, MIR164, MIR1918, MIR1919, MIR394, MIR399, MIR530, MIR5300, MIR5301, MIR6020, MIR6022, MIR8007, MIR8011, MIR8021 and MIR8031, contained only one member. Gao et al. [27] has reported that miRNA families MIR156, MIR159, MIR160, MIR166, MIR167, miRNA171 and MIR398, are ancient, as their orthologs are distributed in Coniferophyta and Embryophyta. On the contrary, six families (MIR162, MIR164, MIR168, MIR172, MIR394 and MIR399) had homologs in angiosperms, idicating that these *L. chinense* miRNA families might have recently evolved.

### Prediction of potentially novel miRNAs and nucleotide bias

**Figure 3:**
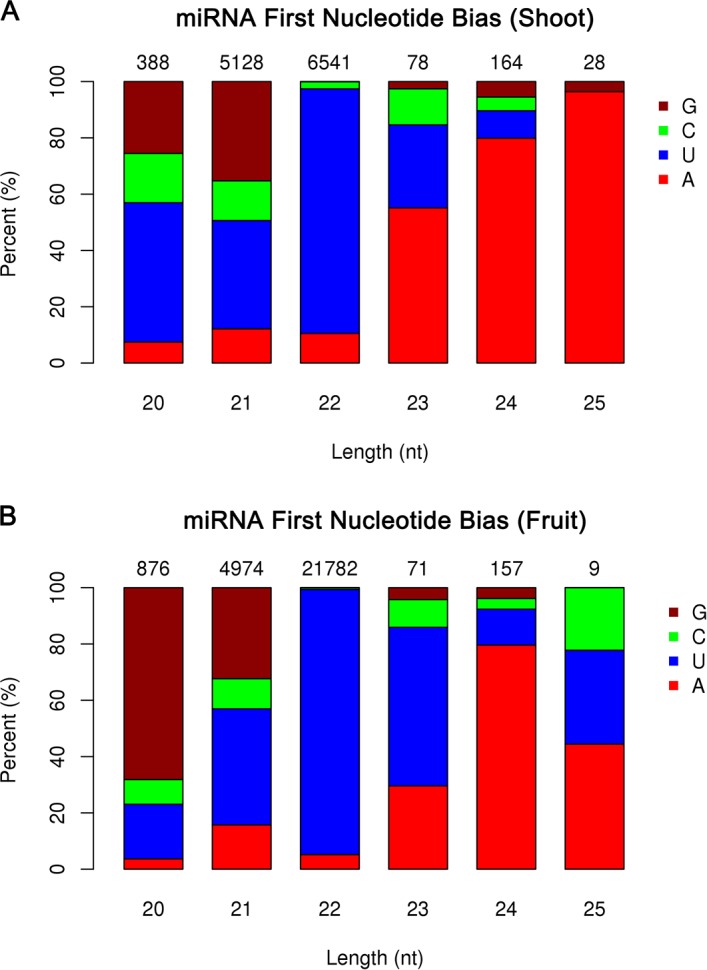
First nucleotide bias of novel miRNA candidates in the *L. chinense* shoot (A) and fruit (B) libraries. The number on top of the bars indicates the number of sequences corresponding to the miRNA length; “0” indicates the absence of sequences of that corresponding length. X-axis, length of miRNAs; Y-axis, representing percent of each nucleotide.

The software programs miREvo and miRDeep2 were used to predict potentially novel miRNAs from the unannotated small-RNA tags by exploring the secondary structure and dicer cleavage site and measuring the minimum free energy (MFE). Custom scripts were used to obtain the identified miRNA counts and base bias on the first position. The number of novel miRNAs was summarized along with the nucleotide bias of the first position of each small RNA of a certain length (Fig. 3). The majority of these novel miRNAs had a length of 22 nt in both of the libraries, and started with a 5’ U. In contrast, for the known miRNAs, the majority had 21 nt in both of the libraries with a 5’ U. A total of 30 novel mature miRNA sequences were obtained, of which 25 and 29 were shoot- and fruit-specific, respectively. In this study, the miRNA precursor length of the putative novel miRNAs ranged from 46 to 295 nt.

### Differentially expressed known and novel miRNAs between the shoot and fruit

To identify miRNAs regulating fruit development, the expression of each miRNA was normalized to transcripts per million (TPM) and compared between the shoot and fruit. Of 60 known mature miRNAs, 14 were only found in the shoot, and 7 were only found in the fruit, with 39 being commonly expressed in both of the tissues. The number of expressed miRNAs varied between the shoot and fruit, with 53 known miRNAs expressing in the shoot and 46 known miRNAs in the fruit (S1 Table). However, this pattern was opposite in the case of novel miRNAs, with 29 novel miRNAs being expressed in the fruit and 25 in the shoot, with 5 fruit-specific and only 1 shoot-specific novel miRNA (Table 3). Moreover, the expression level of miRNAs varied greatly, i.e., the abundance ranged from 24.55 TPM to 63,523 TPM in the shoot and from 27.1 TPM to 693,556 TPM in the fruit. In shoot library, miR166b, miR5301 and miR162 were the most represented families with abundances of 63,523 TPM, 18,269 TPM and 14,610 TPM, respectively. These three miRNA families were also found to be highly represented in fruit, but the top three were miR167, miR166b and miR162 with 693,356 TPM, 160,252 TPM and 75,796 TPM, respectively. Interestingly miR166b was expressed most frequently, ranking first in the shoot and second in the fruit, covering nearly 67% of the differentially expressed known miRNA transcripts in the shoot and fruit libraries (S1 Table). A previous report has identified the rice homeobox-leucine zipper protein HOX32 as the target of miR166. These types of transcription factors are involved in various tissues or different plant developmental stages [28].

**Table 3:** Description of 30 potentially mature novel miRNAs.

**miRNA**	**ML (nt)**	**Sequence**	**PL (nt)**	**miRNA reads**		**Ratio (Shoot/Fruit)**	miRNA*
				**Shoot**	**Fruit**		
LC1	22	uagggcguucggauccuucugc	125	2887	18,833	0.29	Yes
LC11	21	aauccuucugcaauccauaac	144	269	405	1.27	Yes
LC14	21	uagaaagaguuuguaggcgag	286	56	438	0.24	Yes
LC16	22	ucuuaccaauaccucccauucc	94	346	182	3.64	Yes
LC17	22	uccaaucuccucgcccauauuu	76	0	95	-	Yes
LC2	22	uugccaauuccccccauuccga	76	2662	468	10.88	Yes
LC20	21	cgggguauuguaaguggcaga	92	56	84	1.28	No
LC22	24	acaagcucugauaccauguaggaa	104	102	73	2.67	Yes
LC23	21	uccaaauaaaccuccaucgug	82	3	39	0.15	No
LC24	21	uuguugugugauaaauccagg	129	9	9	1.91	No
LC25	21	cauaggauucuugggcaugcu	240	0	18	-	Yes
LC26	22	ucucuuucggucuagaucgucu	295	4	21	0.36	No
LC27	24	acaggcucugauaccaugaaggaa	102	25	15	3.19	No
LC28	21	uuauuaucucagaaagucacu	80	3	0	-	Yes
LC29	21	uuucugguguauagugauaau	93	10	3	6.38	No
LC3	22	uuaaaaaggguuauguaguggc	279	0	1788	-	No
LC30	21	cagcagcgguaggaaugaagc	81	0	6	-	Yes
LC31	21	caaaggccacaagauucacuu	148	5	1	9.56	Yes
LC32	21	auaauacuuggaauaugcccu	222	330	127	4.97	Yes
LC33	24	aaucccgggauuguaguguuauuu	46	11	16	1.32	No
LC34	24	auguugcucggacucuuugaaaau	107	3	5	1.15	No
LC35	21	aaacccucagcgauccauaac	144	0	3	-	Yes
LC36	21	uuugcgugaacacuacagagu	66	17	18	1.81	No
LC37	21	ugucgcaggugacuuucgccc	85	673	404	3.19	No
LC4	20	gaucaugugguagcuucacc	73	85	704	0.23	No
LC40	21	agaaagcaagguuucaggugu	235	8	3	5.10	No
LC41	21	uuaaggcguguagaugugcau	143	245	41	11.43	No
LC46	21	ucggacucuucaaaaauguug	107	1	4	0.48	No
LC6	21	ugggucgcugaaggauugaug	172	465	357	2.49	Yes
LC9	21	uuagauucacgcacaaacuug	75	409	316	2.48	No

ML, mature sequence length; PL, Precursor length; miRNA*, the sequences that can form miRNA::miRNA* with miRNA variants.

Our high-throughput sequencing discovered 73.33% (44 of 60) conserved miRNAs and 60% (18 of 30) novel miRNAs that were significantly differentially expressed in the fruit, either as up- or down-regulating patterns. A total of 12 significantly differentially expressed miRNAs (7 known and 5 novels) were specific to the fruit-tissues, while 15 (14 known and 1 novel) were specific to the shoot tissues. These results suggested that the miRNAome, as well as the small-RNA transcriptome [29], might be very complex, although miRNA sequences are distinctly conserved across plant species [15].

Among the 44 differentially expressed known miRNAs, 17 were up-regulated, and 27 were down-regulated in the fruit significantly at the *P*<0.01 level. In the case of novel miRNAs, the pattern was opposite, as11 were up-regulated, and 7 were down-regulated in the fruit tissues. Of these 62 (44 known and 18 novel) differentially expressed miRNAs, 43 (34 known and 9 novel) demonstrated 3-fold or greater expression changes between the shoot and fruit tissues (Table 4).

**Table 4:** Significantly differentially expressed miRNAs showing 3 or more log2-fold expression changes.

**sRNA**	**Shoot Reads (TPM)**	**Fruit Reads (TPM)**	**log2-fold change**	***P*-value**	**Signature**	**Regulation**	**Sig level**
miR156a	122.78	21,864.83	-7.48	0	TRUE	Down	^**^
miR156f-5p	24.56	20,160.03	-9.68	0	TRUE	Down	^**^
miR156h-3p	0	27.1	-5.76	5.28E-06	TRUE	Down	^**^
miR164a	491.10	19,104.67	-5.28	0	TRUE	Down	^**^
miR167	4468.99	69,355.9	-3.96	0	TRUE	Down	^**^
miR167a	2602.82	36,450.41	-3.80	0	TRUE	Down	^**^
miR171a	3486.80	351.79	3.31	0	TRUE	Up	^**^
miR171b	49.11	0	6.62	3.80E-18	TRUE	Up	^**^
miR171d	245.55	0	8.94	4.08E-64	TRUE	Up	^**^
miR172a	220.99	27.1	3.03	7.09E-75	TRUE	Up	^**^
miR172c-3p	98.22	0	7.62	1.81E-31	TRUE	Up	^**^
miR1918	0	54.12	-6.76	4.98E-11	TRUE	Down	^**^
miR1919–5p	24.56	0	5.62	2.10E-10	TRUE	Up	^**^
miR319	4223.44	270.6	3.96	0	TRUE	Up	^**^
miR319a	4223.44	270.60	3.96	0	TRUE	Up	^**^
miR319b	4223.44	270.60	3.96	0	TRUE	Up	^**^
miR394	6310.60	595.33	3.41	0	TRUE	Up	^**^
miR397a	0	27.06	-5.76	5.28E-06	TRUE	Down	^**^
miR397b	0	135.30	-8.08	2.30E-24	TRUE	Down	^**^
miR398	49.11	4329.67	-6.46	0	TRUE	Down	^**^
miR398a-3p	638.43	6359.20	-3.32	0	TRUE	Down	^**^
miR398b-3p	24.56	0	5.62	2.10E-10	TRUE	Up	^**^
miR530	73.67	0	7.20	4.56E-25	TRUE	Up	^**^
miR5300	0	108.24	-7.76	3.92E-20	TRUE	Down	^**^
miR5303	0	27.06	-5.76	5.28E-06	TRUE	Down	^**^
miR5303c	0	27.06	-5.76	5.28E-06	TRUE	Down	^**^
miR5303f	24.55	0	5.62	2.10E-10	TRUE	Up	^**^
miR6022	73.66	0	7.20	4.56E-25	TRUE	Up	^**^
miR6024	73.66	0	7.20	4.56E-25	TRUE	Up	^**^
miR6024–3p	73.66	0	7.20	4.56E-25	TRUE	Up	^**^
miR6025a	98.22	0	7.62	1.81E-31	TRUE	Up	^**^
miR6025d	98.22	0	7.62	1.81E-31	TRUE	Up	^**^
miR8007a-5p	24.55	0	5.62	2.10E-10	TRUE	Up	^**^
miR8011a-3p	24.55	0	5.62	2.10E-10	TRUE	Up	^**^
LC14	1375.07	11,852.47	-3.11	0	TRUE	Down	^**^
LC17	0	2570.74	-12.33	8.35E-256	TRUE	Down	^**^
LC23	73.66	1055.36	-3.84	3.22E-121	TRUE	Down	^**^
LC25	0	487.09	-9.93	2.36E-70	TRUE	Down	^**^
LC28	73.66	0	7.20	4.56E-25	TRUE	Up	^**^
LC3	0	48,384.06	-16.57	0	TRUE	Down	^**^
LC30	0	162.36	-8.34	1.94E-28	TRUE	Down	^**^
LC35	0	81.18	-7.34	1.05E-15	TRUE	Down	^**^
LC4	2087.16	19,050.55	-3.19	0	TRUE	Down	^**^

TPM, normalized transcripts per million; Signature-TRUE, differentially expressed miRNAs;

*, significant at *P* <0.05;

**, significant at *P* < 0.01.

Dramatic expression differences were also observed in the members of the same miRNA family. For example, miR156a, miR156f-5p and miR156h were expressed at significantly higher levels in the fruit than the shoot with 7.48-, 9.68- and 5.76-fold changes, respectively. Additionally, miR156h-3p was not expressed in the shoot tissues at all. The target genes of these miRNAs are responsible for DNA binding, and have some specific targets that are responsible for the biosynthesis of biochemicals and for cellular functions (S2 Table). Additionally, these 14 up-regulated known miRNAs with a more than 3-fold expression change varied dramatically in *L. chinense* fruit.

### Prediction and annotation of potential target genes of differentially expressed miRNAs

The sequence complementarity between miRNAs and their corresponding target genes offers a straightforward process with which to efficiently search miRNAs target genes [30]. A total of 1139 target genes were found for 90 known and novel miRNAs. A total of 703 target genes were identified for 40 out of the 62 significant differently expressed miRNAs.

The annotation of these target genes was conducted based on GO enrichment and KEGG analyses. The number of target genes for each differentially expressed miRNAs ranged from one to more than one hundred. The highest number of target genes was predicted for miR5303 (142), followed by miR5303g (87) and miR5303 (47). However, the novel miRNA LC35 and miR5300 had only one target gene. Notably, there were 22 significant differentially expressed miRNAs that did not match with any target genes, making it impossible to understand the function of these miRNAs (S6 Table). This phenomenon could be explained by the low expression of target genes or the inefficiency of the cross-species mapping of these miRNAs with the *L. barbarum* transcriptome.

**Figure 4:**
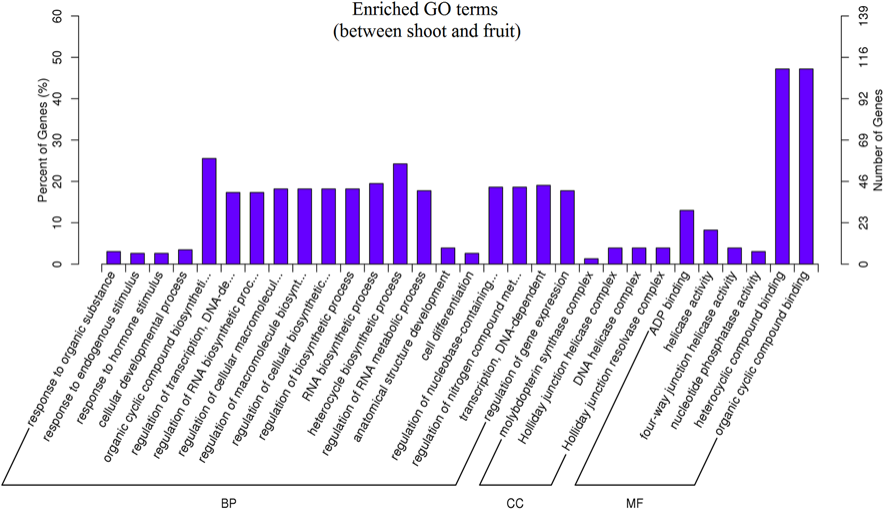
Gene ontology terms and numbers of the predicted target genes for differentially expressed miRNAs. BP, Biological Process; CC, Cellular Component; MF, Molecular Function. Right-hand-side scale, targeted gene numbers corresponding to the GO terms; left-hand-side scale, percent of targeted gene numbers corresponding to the GO terms.

GO (Gene Ontology) categories were assigned for target genes, and 975 putative targets were identified for the top 30 enriched GO categories in terms of ‘biological process’, ‘cellular component’ and ‘molecular function’ (Fig. 4). Based on the biological process, the genes were classified into 20 categories of which the top three over-represented GO terms were “response to organic substances”, “endogenous stimuli” and “hormone stimuli”. The successive analysis revealed that target genes were highly diversified with respect to function. In the case of molecular function, the genes were classified into 6 groups, of which they are mostly involved in “ADP binding”, “helicase activity” and “four-way junction helicase activity”. Based on cellular components, the genes were classified into 4 categories, of which the top three were related to the “molybdopterin synthase complex”, “holiday junction helicase complex” and “DNA helicase complex”. The greatest numbers of genes were involved in “heterocyclic compound binding” (109 genes) and “organic cyclic compound binding” (109 genes) under the molecular function terms. The most over-represented GO term was “ADP binding”, involving 30 genes, followed by “response to organic substances” and “molybdopterin synthase complex”, with 7 and 3 genes, respectively. The significant differences in the categories for cell component occured in the nucleus, chloroplast and plastid, and a striking difference in the biological process was found for transcription regulation, protein modification and photosynthesis. The high frequency of the terms “protein modification” and “transcripts regulation” suggest that miRNAs are closely involved in transcription and translation [31]. Analyses of gene sets for statistically enriched GO terms have shown in S1 Fig.

**Figure 5:**
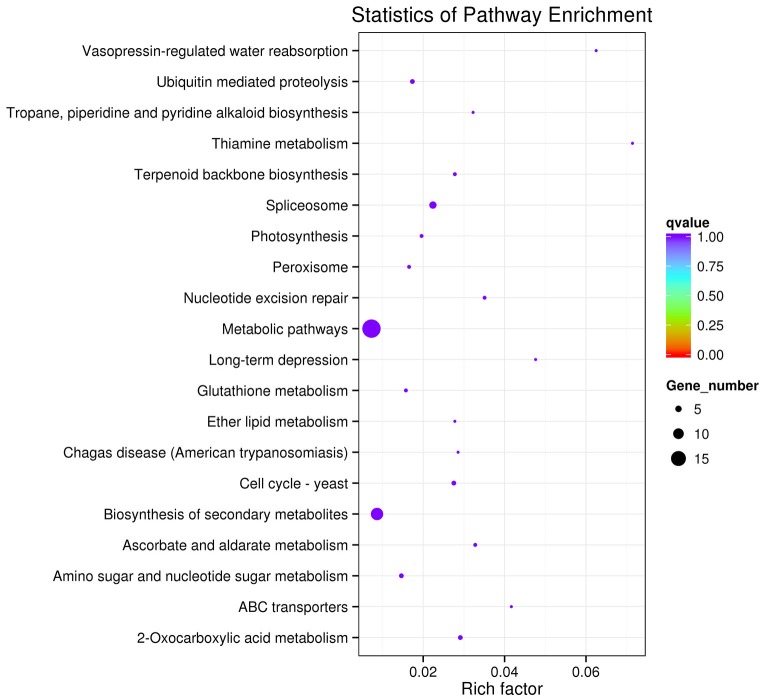
KEGG analysis with the 20 most enriched pathways. The coloring of the q-values indicates the significance of the rich factor; the circle indicates the target genes that are involved, and the size is proportional to the gene number.

Furthermore, a KEGG pathway analysis was performed to illuminate the biological interpretation of these target genes of differentially expressed miRNAs. A total of 75 highly diversified biochemical pathways were involved with identified miRNAs target genes, considering a corrected *P*-value and the number of involved genes. Moreover, the top 20 enriched pathways were discovered with 68 involved genes (Fig. 5). “Metabolic pathways” was the most significantly enriched with respect to the rich factor and gene number (19 genes), followed by “biosynthesis of secondary metabolites” (12 genes) and “spliceosome” (6 genes). S4 Table presented details of these pathways, id name, gene name and hyperlinks.

### Expression validation of sequencing data with qRT-PCR and RLM 5’-RACE

**Figure 6:**
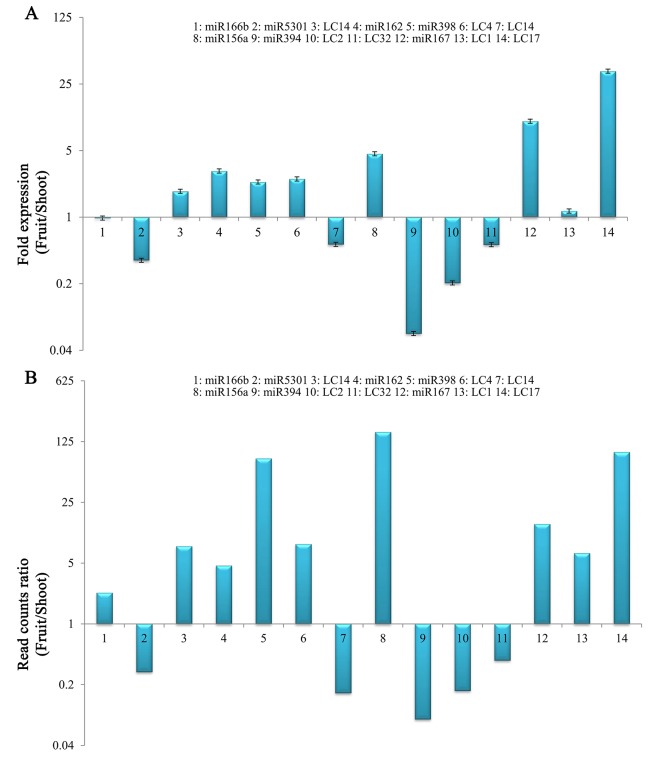
Expression ratios (Fruit/Shoot) of miRNAs in *L. chinense* based on the qRT-PCR (A) and deep sequencing (B) results. X-axis, name of the miRNAs that were selected for qRT-PCR; column above the X-axis, miRNAs that were up-regulated in the fruit; column below the X-axis, miRNAs that were up-regulated in the shoot.

**Figure 7:**
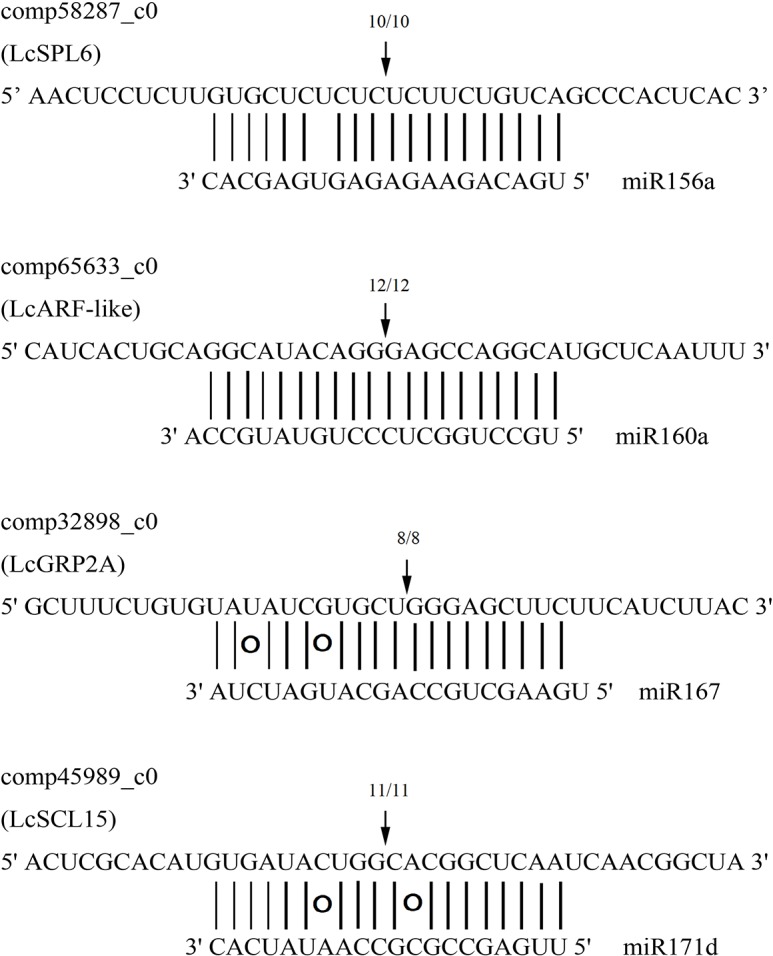
Target validation of known *L. chinense* miRNAs. Arrows showing the 5’-ends of cleavage products.

Quantitative RT-PCR was used to experimentally validate the sequencing data. A total of 14 (7 conserved and 7 novel) miRNAs with different expression levels were randomly selected (Fig. 6). The C_T_ values and RQ values of these miRNAs were obtained, and 12 miRNAs had a similar expression pattern in the fruit and shoot compared with the sequencing results. For example, miR156a was highly expressed in the fruit with 7.48-fold increases compared with the shoot, and a similar result was reflected in the qRT-PCR results, where approximately 7 times greater expression was found in the fruit. However, miR166b and LC14 showed acceptable inconsistency in the expression profile between the sequencing data and qRT–PCR. The possible reasons for this inconsistency might be the difference in the amplification performance of primers or other unknown reasons. The primer designing and their corresponding expression has listed in S5 Table. Additionally, four important targets were validated by modified RLM 5’-RACE with their miRNA cleavage sites (Fig. 7).

## Discussion

The exclusive application of high-throughput sequencing is rapidly replacing the traditional Sanger sequencing platforms for the identification of miRNAs. By now, the most conserved miRNAs have been identified in Arabidopsis, rice and poplar [32]. Recent studies, of non-model plants [33], have reported that the non-conserved and species-specific miRNAs are normally found at a lower abundance than conserved miRNAs, and such a low abundance is difficult to determine efficiently and cost-effectively. However, high-throughput sequencing has been successfully applied for both model plants [29, 34–36] and non-model plants [33, 37, 38], and has also been recognized as a blessing of miRNA identification [39].

### Overview of the deep-sequencing datasets

Length distribution analysis is important to determine the compositional profiling of small RNAs. Normally, miRNA length is 21 nt or 22 nt, whereas, 24 nt in length is considered typical for siRNAs [14]. For both of the libraries, 24-nt small RNA sequences were the most abundant, suggesting that sRNAs of this size class play important roles in *L. chinense*. The same observation was previously reported on *Arabidopsis thaliana* [36], *Citrus sinensis* [40], *Medicago truncatula* [41] etc. However, an opposite scenario was reported in *Pinus contorta*, where 24-nt RNAs constitute only 2.5%, and 21 nt miRNAs are highly enriched up to 50% [27]. Additionally, Lippman et.al [50] and Lu et.al [29, 42] reported the 24-nt sRNAs were predominantly comprised of siRNAs associated with repeats and transposons. In the monocot model plant rice, 24-nt sRNAs comprises approximately 20% while 21-nt sRNAs comprises approximately 10% [37], indicating that a distinct difference exists but, may be not very severe. In this study, 1.36% of the sRNA reads were repeat-associated in the shoot and 1.61% in the fruit libraries. These repeat-associated reads were remarkably more frequent than any other class of sRNA in the two sRNA libraries (Table 2). These results also suggested that the siRNAs that are related to repeat and transposons might have a dominating affiliation both in the shoot and fruit tissues of *L. chinense*. The variation in the length distribution confirmed that the sRNA transcriptomes are complicated and might be dramatically different depending on the evolutionary status of the target species. The results of this study indicate that the MIR166, MIR159, MIR167, MIR162, MIR156 and MIR5301 miRNA families have relatively higher abundance (Fig. 2). Among these families, the highly conserved miRNAs, such as MIR156, MIR159, and MIR166, tended to have greater abundance. However, the non-conserved miRNAs, such as MIR8011, MIR8021, MIR807, MIR1919, had a relatively lower abundance.

There are minimum annotating criteria regarding the biogenesis and expression in which miRNAs can be folded into a stem-loop structure, and mature miRNAs sequences should be detected by northern blotting, qRT-PCR or sequencing analysis [43]. Furthermore, the sequence detection or cloning data of miRNA* biogenesis has been required as a proof of biogenesis because miRNA*s are complementary to miRNAs sequences in the precursor [16, 44]. Among the 30 identified mature novel miRNA candidates, 16 were found with complementary miRNA*s (Table 3). Therefore, these novel mRNAs with miRNA* are likely to be recently evolved (young miRNAs) and species-specific to *L*. *chinense*. The secondary structures of the identified novel miRNAs are listed in S3 Table.

The length of small RNAs in the shoot and fruit libraries was enriched from 21–24 nt, a typical length range in plants (Fig. 1). The small RNAs of 24 nt had a higher expression in both the shoot and fruit sRNA libraries. In addition to the length distribution of small RNAs, there was also a distinct difference between the proportions of various small RNA categories (Table 2). The proportion of rRNA, tRNA, snRNA and snoRNA were higher in the shoot than in the fruit. However, a proportion of the known miRNAs was higher in the shoot, while that of novel miRNAs and that of unannotated reads were higher (82.76%) in the fruit (Table 2). Therefore, the two small RNA libraries exhibited remarkable differences in the small RNA transcriptome in both length distribution pattern and composition, indicating that fruit-related miRNAs are worth more attention in future studies.

### Target gene annotation of fruit-related miRNAs in *L. chinense*

It is possible to identify lowly expressed miRNAs to elucidate the regulation network through high-throughput sequencing. High-throughput technology was first used to identify small RNAs in the fruit, including miRNAs in young green tomatoes, and then extended to different tomato fruit developmental stages [33, 45, 46]. Not surprisingly, a single miRNA can regulate multi-genes, and similarly multiple miRNAs can be used to regulate a single gene, indicating that the miRNA-gene regulation network might be extremely complicated. Li et al. [47] recently identified a set of nine new miRNAs in Solanaceae (tobacco, potato, and tomato), that commonly target R (resistance) genes. Five miRNAs (miR6022–miR6024, miR6026, and miR6027) are expressed during tomato fruit development. In this study, miR5303, miR5303h, miR5303g and miR5303j regulated the same gene, *comp66360_c1*, which is responsible for aminopeptidase activity (S6 Table).

In this study, 7 differentially expressed known miRNAs and 5 novel miRNAs were fruit-specific. A total of 227 target genes were identified for fruit-specific known miRNAs, and one target was found for only one fruit-specific novel miRNAs. Xu et al. [22] reported that miRNA transcriptome regulated lycopene biosynthesis in a red-flesh sweet orange mutant, and three groups of miRNA-target genes were involved in lycopene accumulation. A key gene of the carotenoid biosynthesis pathway is *EY752486*, which is a potential target of csi-miR167. *EY752486* encodes geranylgeranyl pyrophosphate synthase (GGPS) and lycopene β-cyclase (LYCb), which are key enzymes for the production of prerequisite molecules and the conversion of lycopene to downstream cyclic carotenes, respectively [22]. A similar observation was reported in this study, where homologs of miR167, such as miR167a and miR167b, showed a significantly higher expression level in the fruit compared with the shoot, suggesting that miR167 also plays important roles in *L. chinense* fruit. Another interesting finding was that the target gene *APETALA2* (*AP2*), designated as *RAP2.2* in Arabidopsis, interacts with the phyteone synthase (PSY) gene encoding a rate-limiting enzyme for the biosynthesis of carotenoids [48]. The *RAP2.2* transcription factor was also a target gene of the csi-miR172 homologs, miR172 and miR172d-3p in *L. chinense*, and these two miRNAs had a remarkable low expression level in the fruit. Moreover, our GO results identified genes that are involved in photosynthesis or carbon fixation in plastids or chloroplasts. A previous study of the tomato *hp-2* mutant with a high level of lycopene reported that photosynthesis-related genes are consistently up-regulated throughout fruit ripening [49]. Taken together, miRNAs that regulate biological processes, including photosynthesis, transcription regulation and carotenoid biosynthesis, should be given more attention in the study of secondary metabolite accumulation in *L. chinense* fruits.

Further functional analyses of fruit-related miRNAs are also required to elucidate the function of these miRNAs during fruit development. miR156, during ripening regulation in tomato, targets a member of the squamosa-promoter binding protein (SPB) family called CNR [24]. In this study, miR156 was also significantly highly expressed in the fruit. Ethylene is one of the most important phytohormones and plays important roles in the plant life cycle, including seed germination, fruit ripening, and various abiotic stresses [25]. miR159 and miR394 are associated with ethylene response in rice [50]. In *L. chinense*, miR394 exhibited an up-regulated expression in the shoot, and miR159 was highly abundant in both the shoot and the fruit, indicating that ethylene plays regulatory roles in both the vegetative and reproductive phases. miR159 had another target, designated as 1-aminocyclopropane-1-carboxylate synthase, which also plays important roles in ethylene biosynthesis [26]. miR394-targeted F-box proteins are also important in the signal transduction pathways of various plant hormones [51, 52].

To date, fruit-specific miRNA identification has been reported on a very limited scale and includes cloning approaches in tomato [33] and grape [53] and deep sequencing approaches in peach [54], tomato [46] and orange [22]. The results of these reports strongly suggested that the expression and function of fruit-specific miRNAs throughout plant species are lacking, and fruit ripening miRNAs might functions in different organs besides fruits [22].

### Target genes annotation of shoot-specific miRNAs in *L. chinense*

In this study, 15 miRNAs (14 known and 1 novel) were identified as shoot-specific, and a total of 179 targets were discovered for 14 known miRNAs, while the novel miRNA did not have any target. In addition, six known miRNAs were identified with a high expression level in the shoot tissues: miR160a, miR171d, miR172d-3p, miR319, miR394 and miR5301. miR160 regulates auxin response factors (ARFs), which play a central role in the auxin-regulated gene expression of primary response genes [55]. miR319 is a regulator of *Lanceolate* (*La*), which is required for compound leaf development in tomato [56]. miR394 has a regulatory effect in abiotic stress, and miR172 could regulates the expression of the *AP2*-like transcription factor [57]. Moreover, miR172 is a regulator of the developmental phases and specific floral organs in *Arabidopsis thaliana* [57, 58]. Karlova et al. (2013) reported that the *MYB* class of transcription factors is the target of miR5301. Additionally, miR5301 and some of its reported homologs might function in signal transduction or disease resistance [59]. The target genes of shoot-related miRNAs suggest that the miRNAs that are specific to shoot tissues might have broad regulatory functions in transcription factors, abiotic stresses, disease resistance and vegetative development in *L. chinense*.

## Conclusion

This study reported the first set of miRNAs in *L. chinense* and identified 90 conserved and potentially novel miRNAs in shoot and fruit small-RNA libraries, of which 28 had a significantly higher expression level in the fruit than in the shoot of *L. chinense*. Further annotation revealed 30 over-represented GO categories, in which 975 genes were involved in ‘molecular functions’, ‘cellular component’ and ‘biological processes’. Additionally, the 20 most enriched pathways were identified, and 68 target genes were involved. Therefore, this study provides a unique source of miRNAs and target genes for studying biological pathways, especially for the medicinal properties of *L. chinense*.

## Supporting Information

S1 Fig GO terms of statistically enriched target genes in biological system ontology. **A: Biological process; B: Cellular component; C: Molecular function** (GO terms in the square indicate the top enriched terms; red color indicates a higher level of enrichment significance than the pink color).
(TIF)

S2 Fig Plant parts of *Lycium chinense*. **A: Fruits bearing shoot; B: Three developmental stages of fruit and shoot tip.** g, green stage; cb, color breaking stage; mr, mature red stage.
(TIF)

S1 Table All (known and novel) differentially expressed miRNAs in the shoot and fruit tissues of *L. chinense*. (DOCX)

S2 Table GO enrichment results with description of GO accessions and corresponding genes in the *L. chinense* shoot and fruit. (XLSX)

S3 Table The secondary structures of the identified novel miRNAs in *L. chinense*. (PDF)

S4 Table The most enriched pathways that were identified for target genes. (XLSX)

S5 Table The designed primers and candidate list of miRNAs for qRT-PCR with differential expression through deep sequencing. (DOCX)

S6 Table Annotation of target genes of known and novel miRNAs in *L. chinense*. (XLSX)

S7 Table The designed primers for RLM 5’-RACE and targets annotation. (DOCX)

## References

[1] Hou K (1984) A dictionary of the families and genera of Chinese seed plants. Beijing: Science Press.

[2] CAMS (1980) Data book of food ingredients. Beijing: People’s Hygiene Publishing House.

[3] Qian J-Y, Liu D, Huang AG (2004) The efficiency of flavonoids in polar extracts of Lycium chinense Mill fruits as free radical scavenger. Food Chem 87: 283–288. 10.1016/j.foodchem.2003.11.008

[4] Oh Y, Cho W, Im G, Jeong Y, Hwang Y, et al (2012) Anti-inflammatory effect of Lycium Fruit water extract in lipopolysaccharide-stimulated RAW 264.7 macrophage cells. Int Immunopharmacol 13: 181–189. 10.1016/j.intimp.2012.03.020 22483979

[5] Chang R, So K (2008) Use of anti-aging herbal medicine, Lycium barbarum, against aging-associated diseases. What do we know so far? Cell Mol Neurobiol 28: 643–652. 10.1007/s10571-007-9181-x 17710531 PMC11514989

[6] Mao F, Xiao B, Jiang Z, Zhao J, Huang X, et al (2011) Anticancer effect of *Lycium barbarum* polysaccharides on colon cancer cells involves G0/G1 phase arrest. Med Oncol 28: 121–126. 10.1007/s12032-009-9415-5 20066520

[7] Gan L, Zhang S, Yang X, Xu H (2004) Immunomodulation and antitumor activity by a polysaccharide-protein complex from *Lycium barbarum* . Int Immunopharmacol 4: 563–569. 10.1016/j.intimp.2004.01.023 15099534

[8] Luo Q, Cai Y, Yan J, Sun M, Corke H (2004) Hypoglycemic and hypolipidemic effects and antioxidant activity of fruit extracts from *Lycium barbarum* . Life Sci 76: 137–149. 10.1016/j.lfs.2004.04.056 15519360

[9] Carthew R, Sontheimer E (2009) Origins and mechanisms of miRNAs and siRNAs. Cell 136: 642–655. 10.1016/j.cell.2009.01.035 19239886 PMC2675692

[10] Mallory A, Vaucheret H (2006) Functions of microRNAs and related small RNAs in plants. Nat Genet 38: S31–S37. 10.1038/ng0706-850b 16736022

[11] Mosher RA, Lewsey MG, Shivaprasad PV (2010) RNA silencing in plants: Flash report! Silence 1: 13–13. 10.1186/1758-907X-1-13 20591153 PMC2902426

[12] Zhang B, Pan X, Cobb G, Anderson T (2006) Plant microRNA: a small regulatory molecule with big impact. Dev Biol 289: 3–16. 10.1016/j.ydbio.2005.10.036 16325172

[13] Wu L, Zhou H, Zhang Q, Zhang J, Ni F, et al (2010) DNA Methylation Mediated by a MicroRNA Pathway. Mol Cell 38: 465–475. 10.1016/j.molcel.2010.03.008 20381393

[14] Bartel D (2004) MicroRNAs: genomics, biogenesis, mechanism, and function. Cell 116: 281–297. 10.1016/S0092-8674(04)00045-5 14744438

[15] Voinnet O (2009) Origin, Biogenesis, and Activity of Plant MicroRNAs. Cell 136: 669–687. 10.1016/j.cell.2009.01.046 19239888

[16] Jones-Rhoades M, Bartel D, Barte B (2006) MicroRNAs and their regulatory roles in plants. Annu Rev Plant Biol 57: 19–53. 10.1146/annurev.arplant.57.032905.105218 16669754

[17] Llave C, Xie Z, Kasschau K, Carrington J (2002) Cleavage of scarecrow-like mRNA targets directed by a class of Arabidopsis miRNA. Science 297: 2053 10.1126/science.1076311 12242443

[18] Bushati N, Cohen S (2007) MicroRNA functions. Annu Rev Cell Dev Biol 23: 175–205. 10.1146/annurev.cellbio.23.090506.123406 17506695

[19] Sunkar R, Jagadeeswaran G (2008) In silico identification of conserved microRNAs in large number of diverse plant species. BMC Plant Biol 8: 37 10.1186/1471-2229-8-37 18416839 PMC2358906

[20] Song C, Fang J, Li X, Liu H, Chao C (2009) Identification and characterization of 27 conserved microRNAs in citrus. Planta 230: 671–685. 10.1007/s00425-009-0971-x 19585144 PMC2729984

[21] Ana Kozomara SG-J (2013) miRBase: annotating high confidence microRNAs using deep sequencing data. Nucleic Acids Res 42: 68–73. 10.1093/nar/gkt1181 24275495 PMC3965103

[22] Xu Q, Liu Y, Zhu A, Wu X, Ye J, et al (2010) Discovery and comparative profiling of microRNAs in a sweet orange red-flesh mutant and its wild type. BMC Genomics 11: 246 10.1186/1471-2164-11-246 20398412 PMC2864249

[23] Wang X, Xiong A, Yao Q, Zhang Z, Qiao Y (2009) Direct isolation of high-quality low molecular weight RNA of pear peel from the extraction mixture containing nucleic acid. Mol Biotechnol 44: 61–65. 10.1007/s12033-009-9204-6 19669950

[24] Elitzur T, Vrebalov J, Giovannoni JJ, Goldschmidt EE, Friedman H (2010) The regulation of MADS-box gene expression during ripening of banana and their regulatory interaction with ethylene. J Exp Bot 61: 1523–1535. 10.1093/jxb/erq017 20200120 PMC2837265

[25] Bapat VA, Trivedi PK, Ghosh A, Sane VA, Ganapathi TR, et al (2010) Ripening of fleshy fruit: Molecular insight and the role of ethylene. Biotechnol Adv 28: 94–107. 10.1016/j.biotechadv.2009.10.002 19850118

[26] Cara B, Giovannoni JJ (2008) Molecular biology of ethylene during tomato fruit development and maturation. Plant Sci 175: 106–113. 10.1016/j.plantsci.2008.03.021

[27] Gao Z, Shi T, Luo X, Zhang Z, Zhuang W, et al (2012) High-throughput sequencing of small RNAs and analysis of differentially expressed microRNAs associated with pistil development in Japanese apricot. BMC Genomics 13: 371 10.1186/1471-2164-13-371 22863067 PMC3464595

[28] Meijer AH, Scarpella E, Dijk EL, Qin L, Taal AJ, et al (1997) Transcriptional repression by Oshox1, a novel homeodomain leucine zipper protein from rice. Plant J 11: 263–276. 10.1046/j.1365-313X.1997.11020263.x 9076993

[29] Lu C, Tej SS, Luo SJ, Haudenschild CD, Meyers BC, et al (2005) Elucidation of the small RNA component of the transcriptome. Science 309: 1567–1569. 10.1126/science.1114112 16141074

[30] Jones-Rhoades M, Bartel D (2004) Computational identification of plant microRNAs and their targets, including a stress-induced miRNA. Mol Cell 14: 787–799. 10.1016/j.molcel.2004.05.027 15200956

[31] Hobert O (2008) Gene regulation by transcription factors and microRNAs. Science 319: 1785–1786. 10.1126/science.1151651 18369135

[32] Lu SF, Sun YH, Shi R, Clark C, Li LG, et al (2005) Novel and mechanical stress-responsive microRNAs in Populus trichocarpa that are absent from Arabidopsis. Plant Cell 17: 2186–2203. 10.1105/tpc.105.033456 15994906 PMC1182482

[33] Moxon S, Jing R, Szittya G, Schwach F, Rusholme Pilcher R, et al (2008) Deep sequencing of tomato short RNAs identifies microRNAs targeting genes involved in fruit ripening. Genome Res 18: 1602 10.1101/gr.080127.108 18653800 PMC2556272

[34] Rajagopalan R, Vaucheret H, Trejo J, Bartel D (2006) A diverse and evolutionarily fluid set of microRNAs in *Arabidopsis thaliana* . Genes Dev 20: 3407–3425. 10.1101/gad.1476406 17182867 PMC1698448

[35] Lu C, Kulkarni K, Souret FF, MuthuValliappan R, Tej SS, et al (2006) MicroRNAs and other small RNAs enriched in the Arabidopsis RNA-dependent RNA polymerase-2 mutant. Genome Res 16: 1276–1288. 10.1101/gr.5530106 16954541 PMC1581437

[36] Fahlgren N, Howell M, Kasschau K, Chapman E, Sullivan C, et al (2007) High-throughput sequencing of Arabidopsis microRNAs: evidence for frequent birth and death of MIRNA genes. PLoS One 2: e219 10.1371/journal.pone.0000219 17299599 PMC1790633

[37] Morin RD, Aksay G, Dolgosheina E, Ebhardt HA, Magrini V, et al (2008) Comparative analysis of the small RNA transcriptomes of *Pinus contorta* and *Oryza sativa* . Genome Res 18: 571–584. 10.1101/gr.6897308 18323537 PMC2279245

[38] Qiu D, Pan X, Wilson IW, Li F, Liu M, et al (2009) High throughput sequencing technology reveals that the taxoid elicitor methyl jasmonate regulates microRNA expression in Chinese yew (*Taxus chinensis*). Gene 436: 37–44. 10.1016/j.gene.2009.01.006 19393185

[39] Song C, Wang C, Zhang C, Korir N, Yu H, et al (2010) Deep sequencing discovery of novel and conserved microRNAs in trifoliate orange (*Citrus trifoliata*). BMC Genomics 11: 431 10.1186/1471-2164-11-431 20626894 PMC2996959

[40] Xu M, Liu Q, Nisbet A, Cai X, Yan C, et al (2010) Identification and characterization of microRNAs in *Clonorchis sinensis* of human health significance. BMC Genomics 11: 521 10.1186/1471-2164-11-521 20920166 PMC3224684

[41] Szittya G, Moxon S, Santos D, Jing R, Fevereiro M, et al (2008) High-throughput sequencing of *Medicago truncatula* short RNAs identifies eight new miRNA families. BMC Genomics 9: 593 10.1186/1471-2164-9-593 19068109 PMC2621214

[42] Lippman Z, Martienssen R (2004) The role of RNA interference in heterochromatic silencing. Nature 431: 364–370. 10.1038/nature02875 15372044

[43] Ambros V, Bartel B, Bartel DP, Burge CB, Carrington JC, et al (2003) A uniform system for microRNA annotation. RNA 9: 277–279. 10.1261/rna.2183803 12592000 PMC1370393

[44] Meyers B, Axtell M, Bartel B, Bartel D, Baulcombe D, et al (2008) Criteria for annotation of plant microRNAs. Plant Cell 20: 3186–3190. 10.1105/tpc.108.064311 19074682 PMC2630443

[45] Mohorianu I, Schwach F, Jing R, Lopez-Gomollon S, Moxon S, et al (2011) Profiling of short RNAs during fleshy fruit development reveals stage-specific sRNAome expression patterns. Plant J 67: 232–246. 10.1111/j.1365-313X.2011.04586.x 21443685

[46] Zuo J, Zhu B, Fu D, Zhu Y, Ma Y, et al (2012) Sculpting the maturation, softening and ethylene pathway: The influences of microRNAs on tomato fruits. BMC Genomics 13:7 10.1186/1471-2164-13-7 22230737 PMC3266637

[47] Li F, Pignatta D, Bendix C, Brunkard JO, Cohn MM, et al (2012) MicroRNA regulation of plant innate immune receptors. Proc Natl Acad Sci USA 109: 1790–1795. 10.1073/pnas.1118282109 22307647 PMC3277104

[48] Welsch R, Maass D, Voegel T, DellaPenna D, Beyer P (2007) Transcription factor RAP2.2 and its interacting partner SINAT2: Stable elements in the carotenogenesis of Arabidopsis leaves. Plant Physiol 145: 1073–1085. 10.1104/pp.107.104828 17873090 PMC2048778

[49] Kolotilin I, Koltai H, Tadmor Y, Bar-Or C, Reuveni M, et al (2007) Transcriptional profiling of high pigment-2dg tomato mutant links early fruit plastid biogenesis with its overproduction of phytonutrients. Plant physiol 145: 389–401. 10.1104/pp.107.102962 17704236 PMC2048735

[50] Liu Q, Zhang Y-C, Wang C-Y, Luo Y-C, Huang Q-J, et al (2009) Expression analysis of phytohormone-regulated microRNAs in rice, implying their regulation roles in plant hormone signaling. FEBS letters 583: 723–728. 10.1016/j.febslet.2009.01.020 19167382

[51] Wang X, Kong H, Ma H (2009) F-box proteins regulate ethylene signaling and more. Genes Dev 23: 391–396. 10.1101/gad.1781609 19240128

[52] Qiao H, Chang KN, Yazaki J, Ecker JR (2009) Interplay between ethylene, ETP1/ETP2 F-box proteins, and degradation of EIN2 triggers ethylene responses in Arabidopsis. Genes Dev 23: 512–521. 10.1101/gad.1765709 19196655 PMC2648649

[53] Carra A, Mica E, Gambino G, Pindo M, Moser C, et al (2009) Cloning and characterization of small non-coding RNAs from grape. Plant J 59: 750–763. 10.1111/j.1365-313X.2009.03906.x 19453456

[54] Luo X, Gao Z, Shi T, Cheng Z, Zhang Z, et al (2013) Identification of miRNAs and Their Target Genes in Peach (*Prunus persica* L.) Using High-Throughput Sequencing and Degradome Analysis. Plos One 8: e79090 10.1371/journal.pone.0079090 24236092 PMC3827290

[55] Guilfoyle T, Ulmasov T, Hagen G (1998) The ARF family of transcription factors and their role in plant hormone-responsive transcription. CMLS 54: 619–627. 10.1007/s000180050190 9711229 PMC11147363

[56] Ori N, Cohen AR, Etzioni A, Brand A, Yanai O, et al (2007) Regulation of LANCEOLATE by miR319 is required for compound-leaf development in tomato. Nat Genet 39: 787–791. 10.1038/ng2036 17486095

[57] Liu H-H, Tian X, Li Y-J, Wu C-A, Zheng C-C (2008) Microarray-based analysis of stress-regulated microRNAs in *Arabidopsis thaliana* . RNA 14: 836–843. 10.1261/rna.895308 18356539 PMC2327369

[58] Zhu Q-H, Helliwell CA (2011) Regulation of flowering time and floral patterning by miR172. J Exp Bot 62: 487–495. 10.1093/jxb/erq295 20952628

[59] Karlova R, van Haarst JC, Maliepaard C, van de Geest H, Bovy AG, et al (2013) Identification of microRNA targets in tomato fruit development using high-throughput sequencing and degradome analysis. J Exp Bot 64: 1863–1878. 10.1093/jxb/ert049 23487304 PMC3638818

[60] Langmead B, Trapnell C, Pop M, Salzberg SL (2009) Ultrafast and memory-efficient alignment of short DNA sequences to the human genome. Genome Bio 10:R25 10.1186/gb-2009-10-3-r25 19261174 PMC2690996

[61] Benson DA, Karsch-Mizrachi I, Lipman DJ, Ostell J, Sayers EW (2011) GenBank. Nucleic Acids Research 39: D32–D37. 10.1093/nar/gkq1079 21071399 PMC3013681

[62] Griffiths-Jones S, Saini HK, van Dongen S, Enright AJ (2008) miRBase: tools for microRNA genomics. Nucleic Acids Research 36: D154–D158. 10.1093/nar/gkm952 17991681 PMC2238936

[63] Friedlander MR, Mackowiak SD, Li N, Chen W, Rajewsky N (2012) miRDeep2 accurately identifies known and hundreds of novel microRNA genes in seven animal clades. Nucleic Acids Res 40: 37–52. 10.1093/nar/gkr688 21911355 PMC3245920

[64] Wen M, Shen Y, Shi SH, Tang T (2012) miREvo: an integrative microRNA evolutionary analysis platform for next-generation sequencing experiments. BMC Bioinformatics 13: 140–165. 10.1186/1471-2105-13-140 22720726 PMC3410788

[65] Wu H-J, Ma Y-K, Chen T, Wang M, Wang X-J (2012) PsRobot: a web-based plant small RNA meta-analysis toolbox. Nucleic Acids Res 40: 22–28. 10.1093/nar/gks554 22693224 PMC3394341

[66] Zhou L, Chen J, Li Z, Li X, Hu X, et al (2010) Integrated Profiling of MicroRNAs and mRNAs: MicroRNAs Located on Xq27.3 Associate with Clear Cell Renal Cell Carcinoma. Plos One 5: e0015224 10.1371/journal.pone.0015224 PMC3013074 21253009

[67] Storey JD (2003) The positive false discovery rate: A Bayesian interpretation and the q-value. Ann Stat 31: 2013–2035. 10.1214/aos/1074290335

[68] Design R-TPP (2005) Facile means for quantifying microRNA expression by real-time PCR. Biotechniques 39: 519–525. 10.2144/000112010 16235564

[69] Livak KJ, Schmittgen TD (2001) Analysis of Relative Gene Expression Data Using Real-Time Quantitative PCR and the 2−ΔΔCT Method. Methods 25: 402–408. 10.1006/meth.2001.1262 11846609

[70] Young MD, Wakefield MJ, Smyth GK, Oshlack A (2010) Gene ontology analysis for RNA-seq: accounting for selection bias. Genome Biol 11:R14 10.1186/gb-2010-11-2-r14 20132535 PMC2872874

[71] Mao XZ, Cai T, Olyarchuk JG, Wei LP (2005) Automated genome annotation and pathway identification using the KEGG Orthology (KO) as a controlled vocabulary. Bioinformatics 21: 3787–3793. 10.1093/bioinformatics/bti430 15817693

