# Supplementary figures and images for: Identification of MicroRNAs and Target Genes in the Fruit and Shoot Tip of Lycium chinense: A Traditional Chinese Medicinal Plant
Source: PLoS One. 2015 Jan 14;10(1):e0116334. doi: 10.1371/journal.pone.0116334 (PMC4294688; doi:10.1371/journal.pone.0116334)

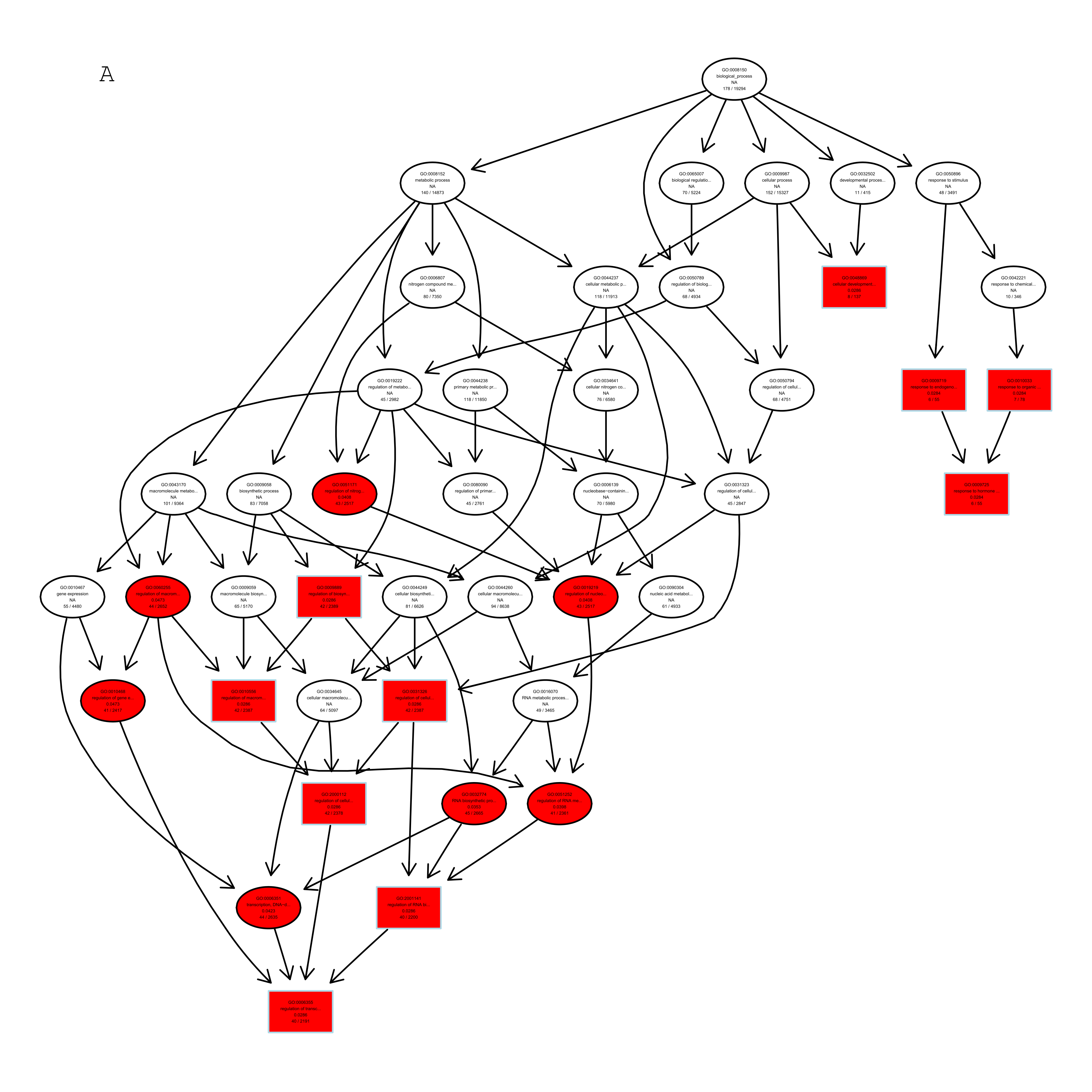

Supplement: S1 Fig — A: Biological process; B: Cellular component; C: Molecular function (GO terms in the square indicate the top enriched terms; red color indicates a higher level of enrichment significance than the pink color). (TIF) [file pone.0116334.s001.tif]

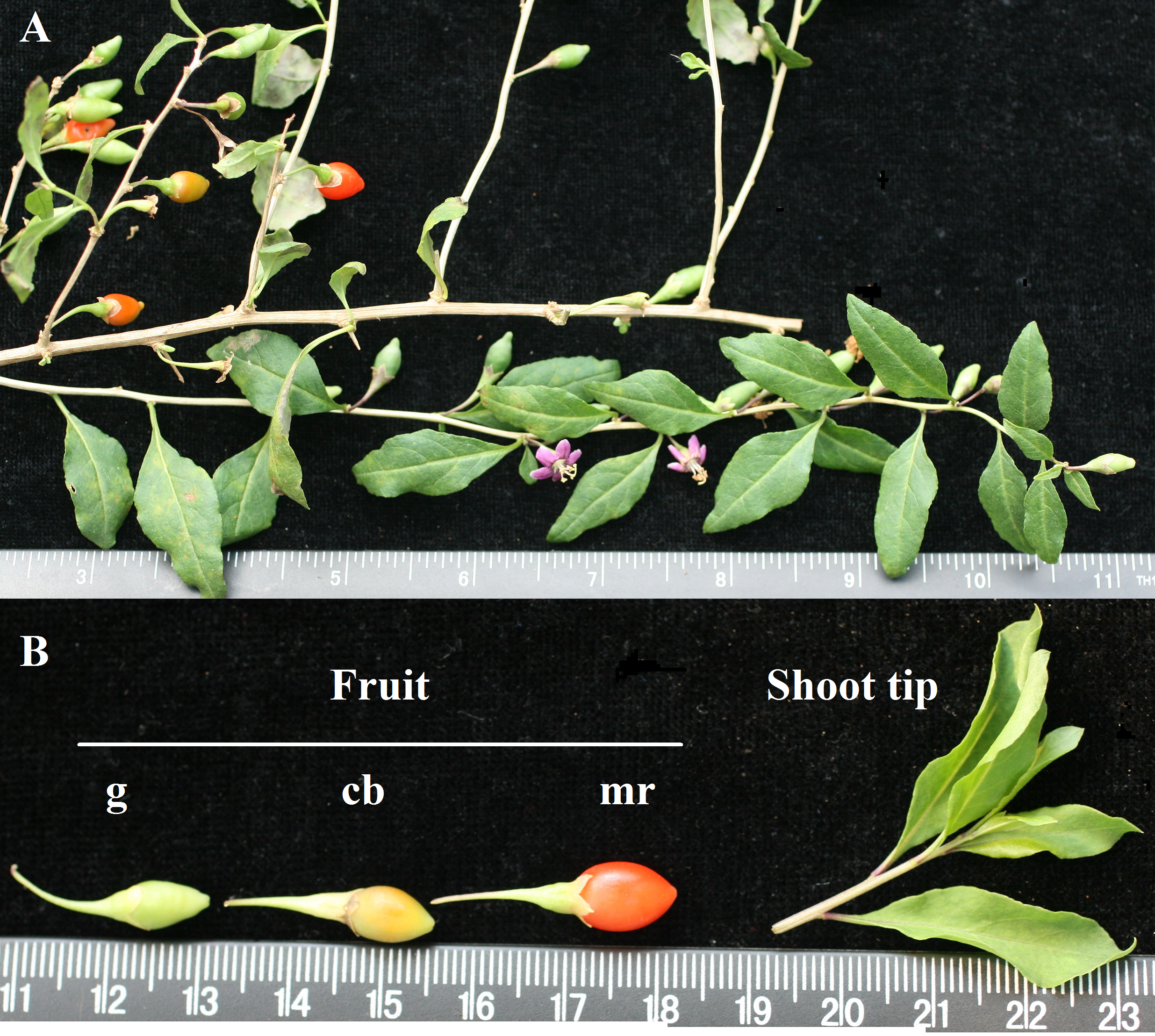

Supplement: S2 Fig — A: Fruits bearing shoot; B: Three developmental stages of fruit and shoot tip. g, green stage; cb, color breaking stage; mr, mature red stage. (TIF) [file pone.0116334.s002.tif]
