# Supplementary material for: Identification of MicroRNAs and Target Genes in the Fruit and Shoot Tip of Lycium chinense: A Traditional Chinese Medicinal Plant
Source: PLoS One. 2015 Jan 14;10(1):e0116334. doi: 10.1371/journal.pone.0116334 (PMC4294688; doi:10.1371/journal.pone.0116334)
Supplement: S1 Table — (DOCX) [file pone.0116334.s003.docx]

**Table S1-A: Sixty conserved known miRNAs in *L*. *chinense* fruit and shoot libraries with their expression levels**

| **Family** | **Members** | **Read counts** | | | **Ratio (shoot/fruit)** |
| --- | --- | --- | --- | --- | --- |
|  |  | **Total** | **Shoot** | **Fruit** |  |
| MIR156 | miR156a | 814 | 5 | 808 | 0.00 |
|  | miR156d | 43 | 27 | 16 | 1.69 |
|  | miR156f-5p | 746 | 1 | 745 | 0.00 |
|  | miR156g | 43 | 27 | 16 | 1.69 |
|  | miR156h-3p | 813 | 0 | 1 | - |
| MIR159 | miR159 | 10189 | 5813 | 4376 | 1.33 |
| MIR160 | miR160a | 30 | 26 | 4 | 6.50 |
| MIR162 | miR162 | 3397 | 595 | 2801 | 0.21 |
|  | miR162a-5p | 3400 | 3 | 1 | 3.00 |
| MIR164 | miR164a | 726 | 20 | 706 | 0.03 |
| MIR166 | miR166a | 14806 | 7007 | 7798 | 0.90 |
|  | miR166a-5p | 13192 | 328 | 555 | 0.59 |
|  | miR166b | 8510 | 2587 | 5922 | 0.44 |
| MIR167 | miR167 | 2746 | 182 | 2563 | 0.07 |
|  | miR167a | 106 | 106 | 1347 | 0.08 |
| MIR168 | miR168a | 531 | 187 | 343 | 0.55 |
|  | miR168d | 129 | 49 | 79 | 0.62 |
| MIR171 | miR171a | 155 | 142 | 13 | 10.92 |
|  | miR171b | 2 | 2 | 0 | - |
|  | miR171d | 10 | 10 | 0 | - |
| MIR172 | miR172a | 57 | 50 | 7 | 7.14 |
|  | miR172a | 10 | 9 | 1 | 9.00 |
|  | miR172c-3p | 4 | 4 | 0 | - |
|  | miR172d-3p | 44 | 37 | 7 | 5.29 |
|  | miR172j | 8 | 5 | 3 | 1.67 |
| MIR1918 | miR1918 | 2 | 0 | 2 | - |
| MIR1919 | miR1919-5p | 1 | 1 | 0 | - |
| MIR319 | miR319 | 182 | 172 | 10 | 17.20 |
|  | miR319a | 182 | 172 | 10 | 17.20 |
|  | miR319b | 182 | 172 | 10 | 17.20 |
| MIR394 | miR394 | 279 | 257 | 22 | 11.68 |
| MIR397 | miR397a | 1 | 0 | 1 | - |
|  | miR397b | 5 | 0 | 5 | - |
| MIR398 | miR398 | 239 | 2 | 160 | 0.01 |
|  | miR398a-3p | 261 | 26 | 235 | 0.11 |
|  | miR398b-3p | 1 | 1 | 0 | - |
| MIR399 | miR399 | 8 | 1 | 7 | 0.14 |
| MIR482 | miR482a | 11 | 4 | 7 | 0.57 |
|  | miR482c | 767 | 479 | 282 | 1.70 |
| MIR530 | miR530 | 3 | 3 | 0 | - |
| MIR5300 | miR5300 | 4 | 0 | 4 | - |
| MIR5301 | miR5301 | 7416 | 744 | 210 | 3.54 |
| MIR5303 | miR5303 | 39 | 0 | 1 | - |
|  | miR5303c | 9 | 0 | 1 | - |
|  | miR5303f | 1 | 1 | 0 | - |
|  | miR5303g | 12 | 7 | 3 | 2.33 |
|  | miR5303h | 11 | 4 | 4 | 1.00 |
|  | miR5303j | 7 | 3 | 4 | 0.75 |
| MIR6020 | miR6020a-5p | 53 | 34 | 19 | 1.79 |
| MIR6022 | miR6022 | 625 | 3 | 0 | - |
| MIR6024 | miR6024 | 3 | 3 | 0 | - |
|  | miR6024-3p | 3 | 3 | 0 | - |
| MIR6025 | miR6025a | 5 | 4 | 0 | - |
|  | miR6025d | 4 | 4 | 0 | - |
| MIR7997 | miR7997a | 42 | 4 | 9 | 0.44 |
|  | miR7997c | 123 | 29 | 57 | 0.51 |
| MIR8007 | miR8007a-5p | 6 | 1 | 0 | - |
| MIR8011 | miR8011a-3p | 1 | 1 | 0 | - |
| MIR8021 | miR8021 | 3 | 1 | 1 | 1.00 |
| MIR8031 | miR8039 | 11 | 7 | 3 | 2.33 |

**Table S1-B: All (known and novel) differentially expressed miRNAs in shoot and fruit tissues of *L. chinense***

| **Sl. No.** | **sRNA** | **Shoot TPM** | **Fruit TPM** | **log2.Fold change** | ***P*-value** | **q.value** | **Signature** |
| --- | --- | --- | --- | --- | --- | --- | --- |
| 1 | LC1 | 70889.9 | 509629.2 | -2.8458 | 0 | 0 | TRUE |
| 2 | LC14 | 1375.07 | 11852.47 | -3.1076 | 0 | 0 | TRUE |
| 3 | LC16 | 8495.1 | 4925.01 | 0.78666 | 0 | 0 | FALSE |
| 4 | LC2 | 65365.06 | 12664.29 | 2.3678 | 0 | 0 | TRUE |
| 5 | LC3 | 0 | 48384.06 | -16.562 | 0 | 0 | TRUE |
| 6 | LC32 | 8103.10 | 3436.67 | 1.2375 | 0 | 0 | TRUE |
| 7 | LC37 | 16525.43 | 10932.42 | 0.59608 | 0 | 0 | FALSE |
| 8 | LC4 | 2087.16 | 19050.55 | -3.1902 | 0 | 0 | TRUE |
| 9 | LC41 | 6015.94 | 1109.47 | 2.4389 | 0 | 0 | TRUE |
| 10 | LC6 | 11418.01 | 9660.57 | 0.24113 | 0 | 0 | FALSE |
| 11 | LC9 | 10042.94 | 8551.1 | 0.232 | 0 | 0 | FALSE |
| 12 | miR156a | 122.7743 | 21864.83 | -7.4765 | 0 | 0 | TRUE |
| 13 | miR164a | 491.1 | 19104.67 | -5.2818 | 0 | 0 | TRUE |
| 14 | miR167a | 2602.81 | 36450.41 | -3.8078 | 0 | 0 | TRUE |
| 15 | miR172a | 1227.74 | 189.42 | 2.6963 | 0 | 0 | TRUE |
| 16 | miR319a | 4223.43 | 270.60 | 3.9642 | 0 | 0 | TRUE |
| 17 | miR394 | 6310.60 | 595.33 | 3.406 | 0 | 0 | TRUE |
| 18 | miR398 | 49.11 | 4329.67 | -6.4621 | 0 | 0 | TRUE |
| 19 | miR159 | 142737.4 | 118416.5 | 0.26949 | 0 | 0 | FALSE |
| 20 | miR162 | 14610.15 | 75796.29 | -2.3752 | 0 | 0 | TRUE |
| 21 | miR166a | 172056 | 211017.3 | -0.29448 | 0 | 0 | FALSE |
| 22 | miR167 | 4468.99 | 69355.9 | -3.956 | 0 | 0 | TRUE |
| 23 | miR171a | 3486.8 | 351.78 | 3.3091 | 0 | 0 | TRUE |
| 24 | miR319 | 4223.43 | 270.60 | 3.9642 | 0 | 0 | TRUE |
| 25 | miR5301 | 18268.82 | 5682.69 | 1.6847 | 0 | 0 | TRUE |
| 26 | miR156f-5p | 24.55 | 20160.03 | -9.6813 | 0 | 0 | TRUE |
| 27 | miR166b | 63523.44 | 160251.9 | -1.335 | 0 | 0 | TRUE |
| 28 | miR319b | 4223.43 | 270.60 | 3.9642 | 0 | 0 | TRUE |
| 29 | miR398a-3p | 638.42 | 6359.20 | -3.3163 | 0 | 0 | TRUE |
| 30 | stu-miR482c | 11761.78 | 7631.04 | 0.62415 | 0 | 0 | FALSE |
| 31 | miR172d-3p | 908.53 | 189.42 | 2.2619 | 1.58E-260 | 4.59E-260 | TRUE |
| 32 | LC17 | 0 | 2570.74 | -12.328 | 8.35E-256 | 2.35E-255 | TRUE |
| 33 | LC22 | 2504.6 | 1975.41 | 0.34242 | 9.34E-253 | 2.55E-252 | FALSE |
| 34 | miR160a | 638.42 | 108.24 | 2.5603 | 2.05E-196 | 5.42E-196 | TRUE |
| 35 | LC23 | 73.66 | 1055.35 | -3.8406 | 3.22E-121 | 8.28E-121 | TRUE |
| 36 | miR6020a-5p | 834.86 | 514.14 | 0.69936 | 1.49E-117 | 3.73E-117 | FALSE |
| 37 | miR156g | 662.98 | 432.96 | 0.61471 | 1.13E-87 | 2.72E-87 | FALSE |
| 38 | miR156a | 662.98 | 432.96 | 0.61471 | 1.13E-87 | 2.72E-87 | FALSE |
| 39 | LC27 | 613.87 | 405.90 | 0.59679 | 4.00E-80 | 9.22E-80 | FALSE |
| 40 | miR172a | 220.1 | 27.06 | 3.0297 | 7.09E-75 | 1.59E-74 | TRUE |
| 41 | LC25 | 0 | 487.08 | -9.928 | 2.36E-70 | 5.19E-70 | TRUE |
| 42 | miR171d | 245.54 | 0 | 8.9399 | 4.08E-64 | 8.74E-64 | TRUE |
| 43 | LC11 | 6605.25 | 10959.48 | -0.73049 | 3.27E-59 | 6.85E-59 | FALSE |
| 44 | LC29 | 245.54 | 81.18 | 1.5968 | 2.36E-58 | 4.83E-58 | TRUE |
| 45 | LC40 | 196.43 | 81.18 | 1.2749 | 6.36E-41 | 1.27E-40 | TRUE |
| 46 | LC31 | 122.77 | 27.06 | 2.1818 | 4.42E-36 | 8.65E-36 | TRUE |
| 47 | miR5303g | 171.88 | 81.18 | 1.0822 | 1.26E-32 | 2.39E-32 | TRUE |
| 48 | miR8039 | 171.88 | 81.18 | 1.0822 | 1.26E-32 | 2.39E-32 | TRUE |
| 49 | miR6025a | 98.22 | 0 | 7.6179 | 1.81E-31 | 3.26E-31 | TRUE |
| 50 | miR6025d | 98.2 | 0 | 7.6179 | 1.81E-31 | 3.26E-31 | TRUE |
| 51 | miR172c-3p | 98.2 | 0 | 7.6179 | 1.81E-31 | 3.26E-31 | TRUE |
| 52 | LC26 | 98.2 | 568.26 | -2.5325 | 7.11E-30 | 1.23E-29 | TRUE |
| 53 | LC30 | 0 | 162.36 | -8.3431 | 1.94E-28 | 3.29E-28 | TRUE |
| 54 | LC28 | 73.66 | 0 | 7.2029 | 4.56E-25 | 7.32E-25 | TRUE |
| 55 | miR6024 | 73.66 | 0 | 7.2029 | 4.56E-25 | 7.32E-25 | TRUE |
| 56 | miR530 | 73.66 | 0 | 7.2029 | 4.56E-25 | 7.32E-25 | TRUE |
| 57 | miR6022 | 73.66 | 0 | 7.2029 | 4.56E-25 | 7.32E-25 | TRUE |
| 58 | miR6024-3p | 73.66 | 0 | 7.2029 | 4.56E-25 | 7.32E-25 | TRUE |
| 59 | miR397 | 0 | 135.30 | -8.08 | 2.30E-24 | 3.50E-24 | TRUE |
| 60 | miR166a-5p | 8053.99 | 15018.54 | -0.89897 | 2.99E-21 | 4.48E-21 | FALSE |
| 61 | LC36 | 417.43 | 487.08 | -0.22264 | 3.93E-20 | 5.70E-20 | FALSE |
| 62 | miR5300 | 0 | 108.24 | -7.7581 | 3.92E-20 | 5.78E-20 | TRUE |
| 63 | miR171b | 49.11 | 0 | 6.6179 | 3.80E-18 | 5.43E-18 | TRUE |
| 64 | miR162a-5p | 73.66 | 27.06 | 1.4448 | 1.33E-17 | 1.87E-17 | TRUE |
| 65 | miR172j | 122.77 | 81.18 | 0.59679 | 2.34E-17 | 3.24E-17 | FALSE |
| 66 | LC35 | 0 | 81.18 | -7.3431 | 1.05E-15 | 1.43E-15 | TRUE |
| 67 | miR399 | 24.55 | 189.42 | -2.9475 | 4.59E-15 | 6.16E-15 | TRUE |
| 68 | LC20 | 1375.07 | 2273.07 | -0.72514 | 7.10E-14 | 9.40E-14 | FALSE |
| 69 | LC24 | 220.99 | 243.54 | -0.14018 | 4.90E-13 | 6.39E-13 | FALSE |
| 70 | miR1918 | 0 | 54.12 | -6.7581 | 4.98E-11 | 6.40E-11 | TRUE |
| 71 | miR1919-5p | 24.55 | 0 | 5.6179 | 2.10E-10 | 2.59E-10 | TRUE |
| 72 | miR398b-3p | 24.55 | 0 | 5.6179 | 2.10E-10 | 2.59E-10 | TRUE |
| 73 | miR5303f | 24.55 | 0 | 5.6179 | 2.10E-10 | 2.59E-10 | TRUE |
| 74 | miR8007a-5p | 24.55 | 0 | 5.6179 | 2.10E-10 | 2.59E-10 | TRUE |
| 75 | miR8011a-3p | 24.55 | 0 | 5.6179 | 2.10E-10 | 2.59E-10 | TRUE |
| 76 | miR168d | 1203.18 | 2137.78 | -0.82925 | 5.34E-07 | 6.33E-07 | FALSE |
| 77 | miR5303h | 98.22 | 108.24 | -0.14018 | 1.45E-06 | 1.69E-06 | FALSE |
| 78 | miR397 | 0 | 27.06 | -5.7581 | 5.28E-06 | 5.97E-06 | TRUE |
| 79 | miR5303 | 0 | 27.06 | -5.7581 | 5.28E-06 | 5.97E-06 | TRUE |
| 80 | miR156h-3p | 0 | 27.06 | -5.7581 | 5.28E-06 | 5.97E-06 | TRUE |
| 81 | miR5303c | 0 | 27.06 | -5.7581 | 5.28E-06 | 5.97E-06 | TRUE |
| 82 | LC46 | 24.55 | 108.24 | -2.1402 | 0.000107 | 0.000117 | TRUE |
| 83 | LC33 | 270.10 | 432.96 | -0.68074 | 0.00021 | 0.000228 | FALSE |
| 84 | miR5303j | 73.66 | 108.24 | -0.55521 | 0.012587 | 0.013486 | FALSE |
| 85 | miR168a | 4591.76 | 9281.73 | -1.0153 | 0.01275 | 0.0135 | FALSE |
| 86 | miR8021 | 24.55 | 27.06 | -0.14018 | 0.015991 | 0.016734 | FALSE |
| 87 | miR7997a | 98.22 | 243.54 | -1.3101 | 0.13974 | 0.14456 | FALSE |
| 88 | LC34 | 73.66 | 135.30 | -0.87714 | 0.31381 | 0.32094 | FALSE |
| 89 | miR482a | 98.22 | 189.42 | -0.94753 | 0.44695 | 0.45198 | FALSE |
| 90 | miR7997c | 712.09 | 1542.44 | -1.1151 | 0.52026 | 0.52026 | FALSE |
