# Supplementary material for: Identification of MicroRNAs and Target Genes in the Fruit and Shoot Tip of Lycium chinense: A Traditional Chinese Medicinal Plant
Source: PLoS One. 2015 Jan 14;10(1):e0116334. doi: 10.1371/journal.pone.0116334 (PMC4294688; doi:10.1371/journal.pone.0116334)
Supplement: S3 Table — (PDF) [file pone.0116334.s005.pdf]

Table S3: List of secondary structures of identified novel miRNAs in *Lycium chinense*.

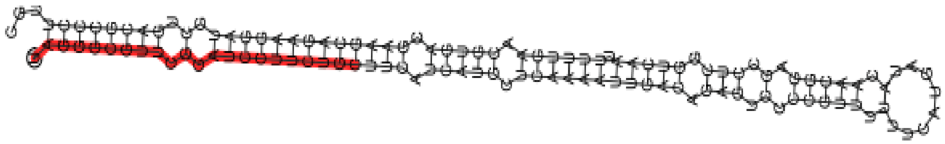

LC1

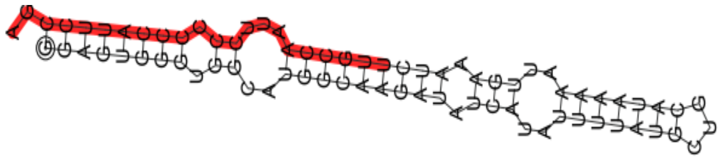

LC2

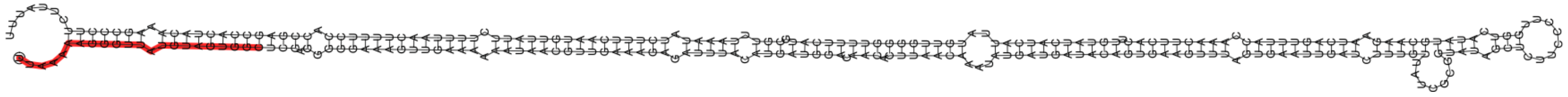

LC3

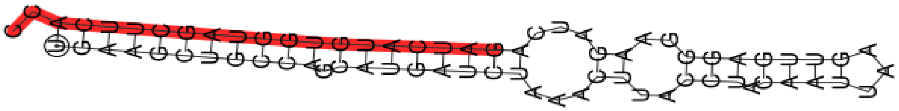

LC4

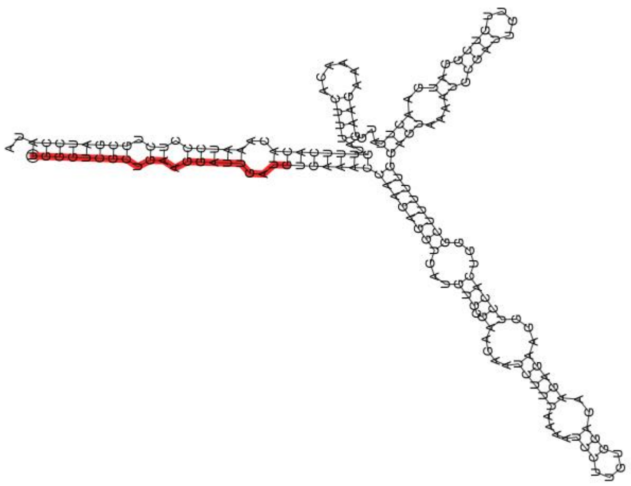

LC6

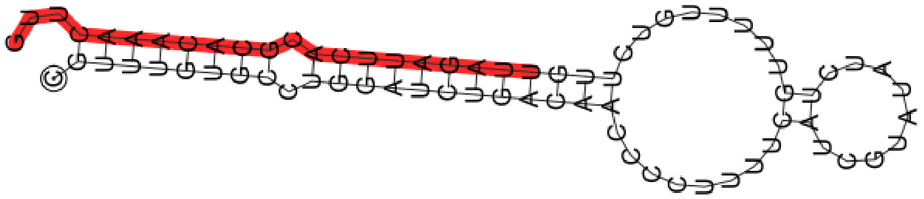

LC9

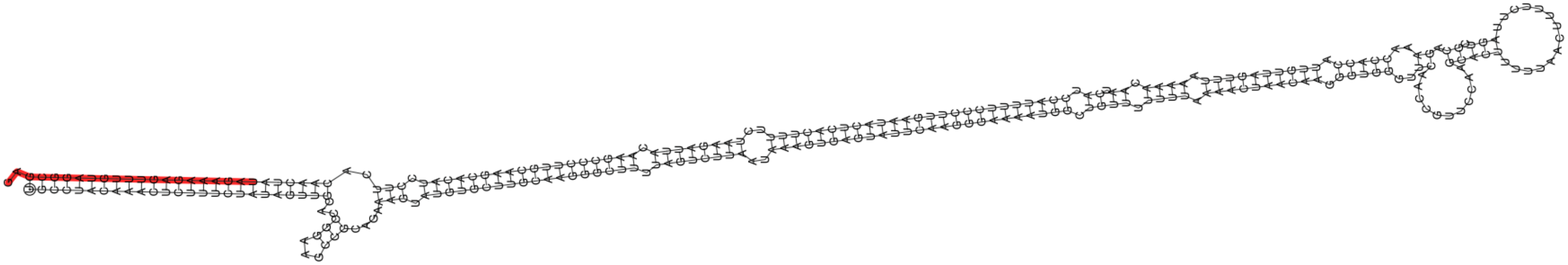

LC14

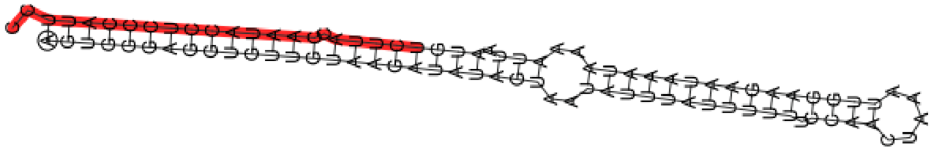

LC16

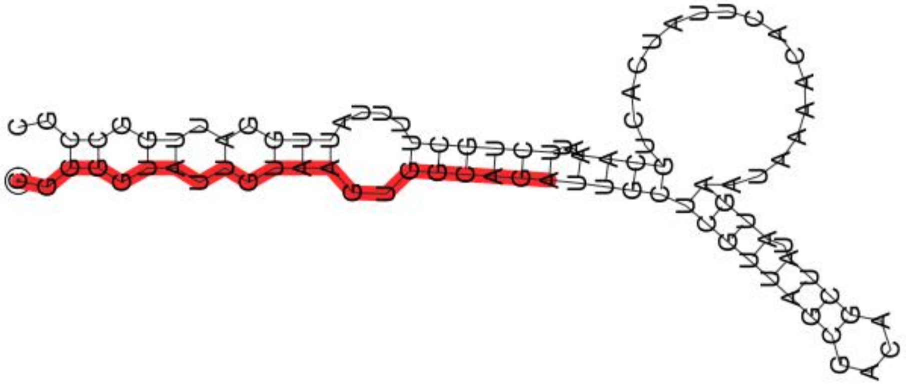

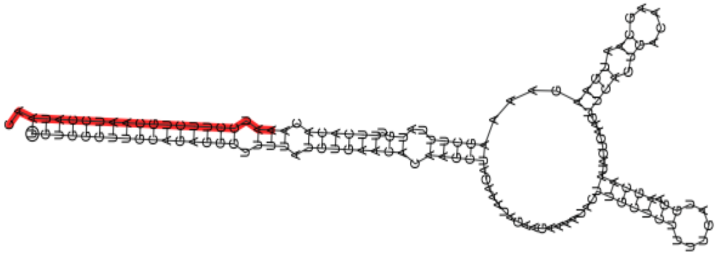

LC11

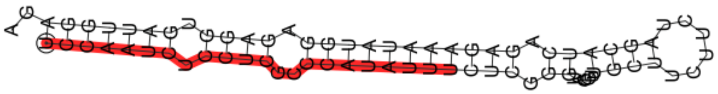

LC17

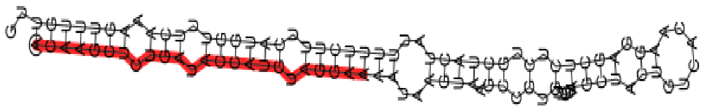

LC22

LC20

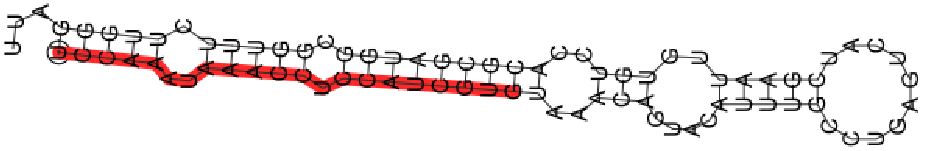

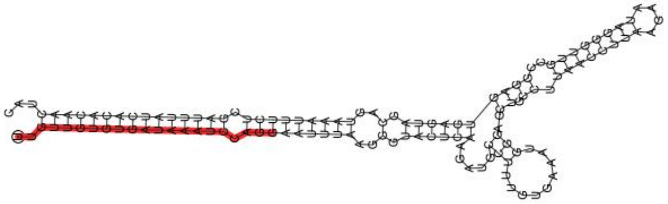

LC23

LC24

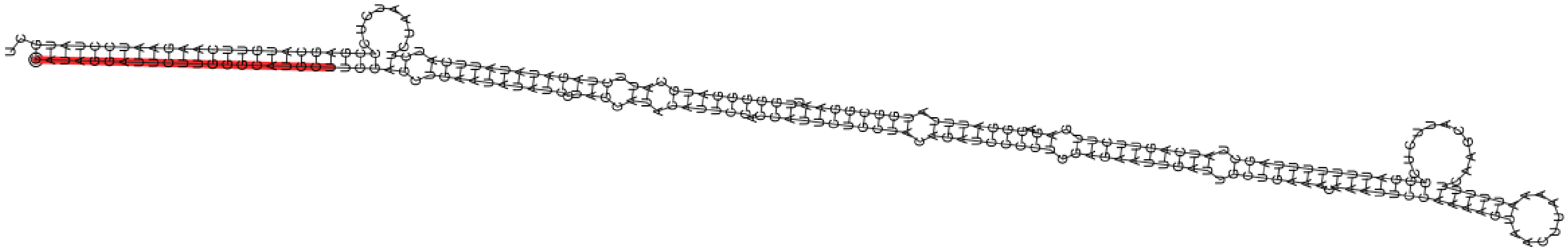

LC25

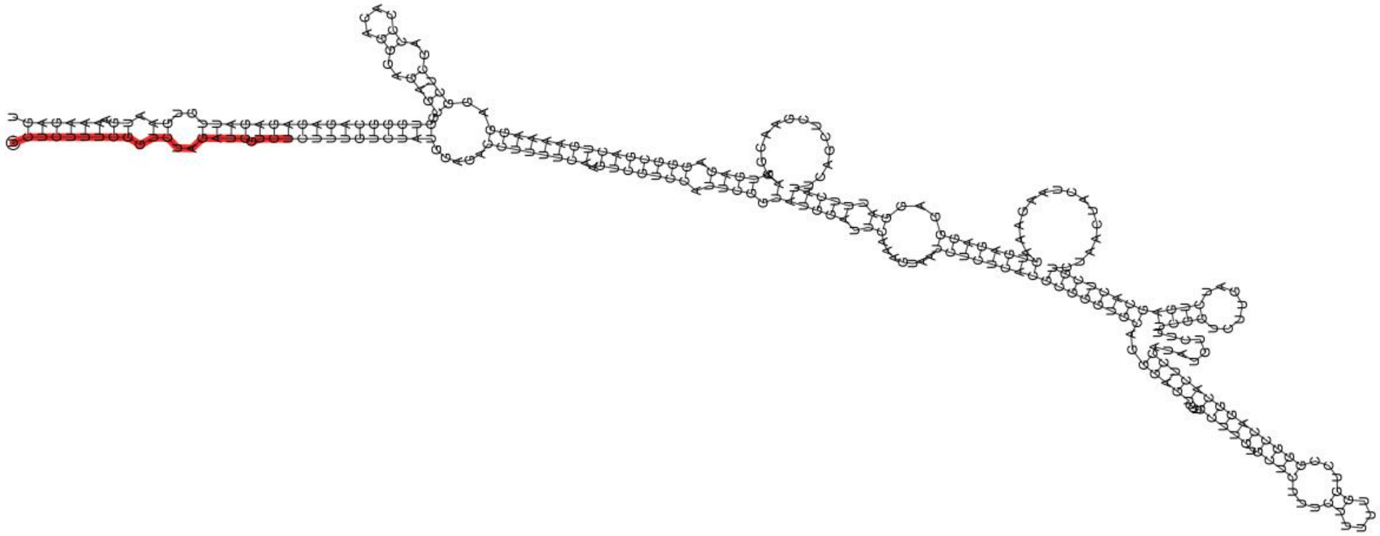

LC26

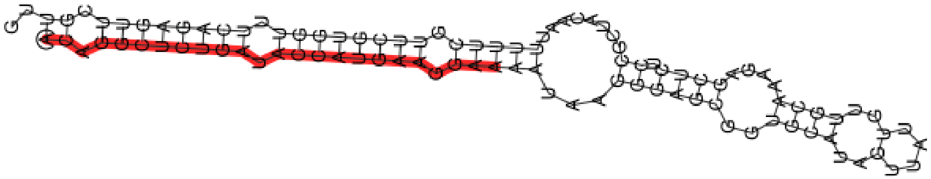

LC27

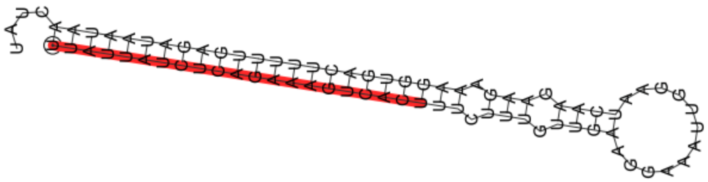

LC28

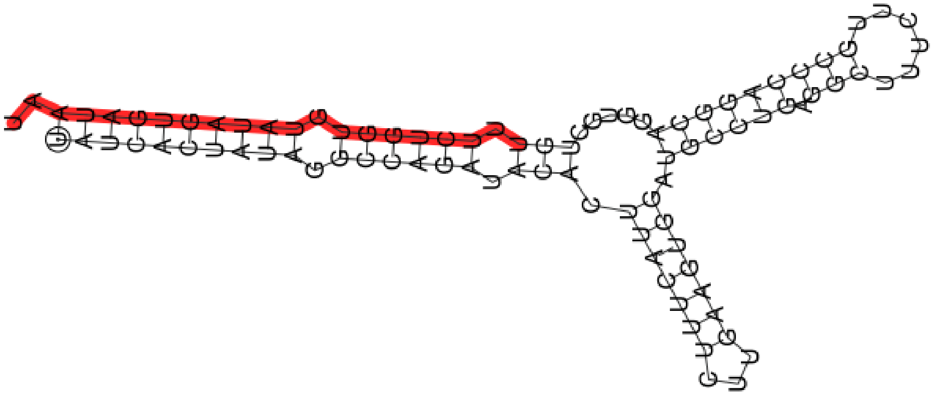

LC29

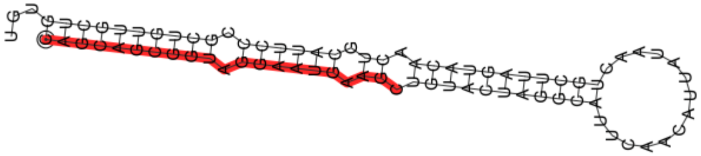

LC30

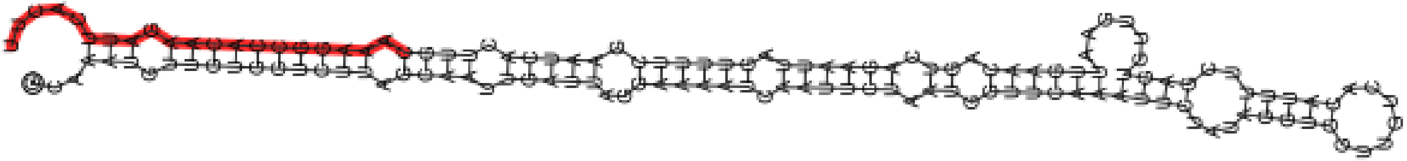

LC31

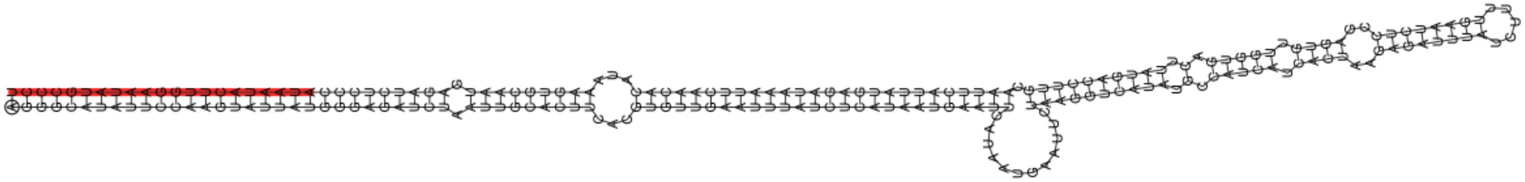

LC32

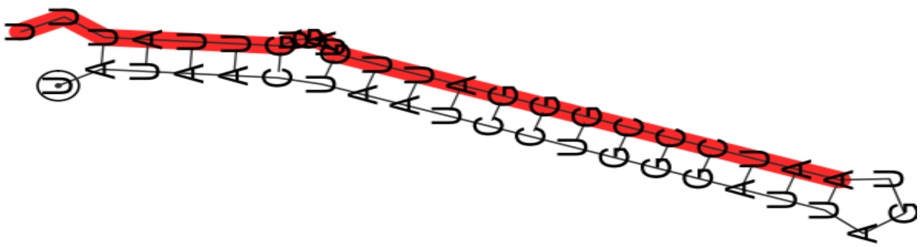

LC33

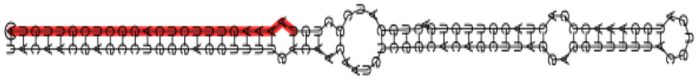

LC34

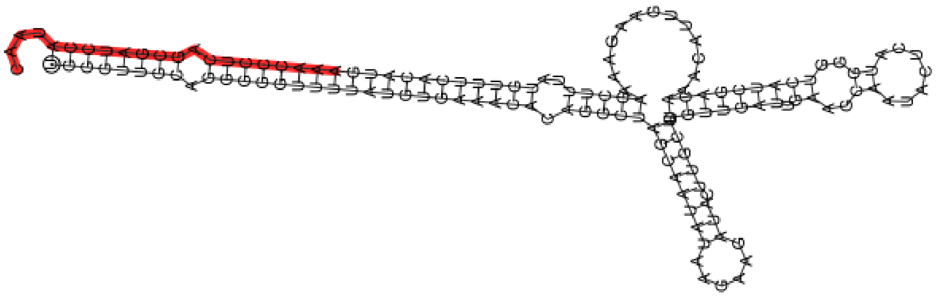

LC35

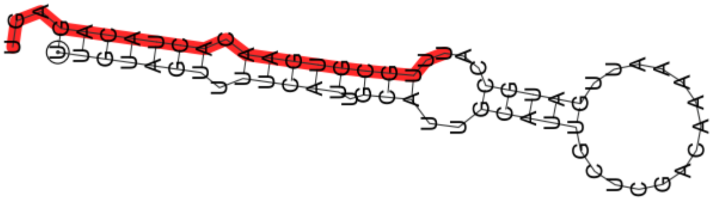

LC36

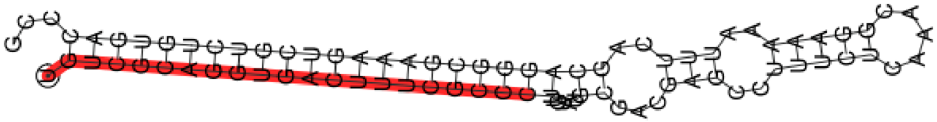

LC37

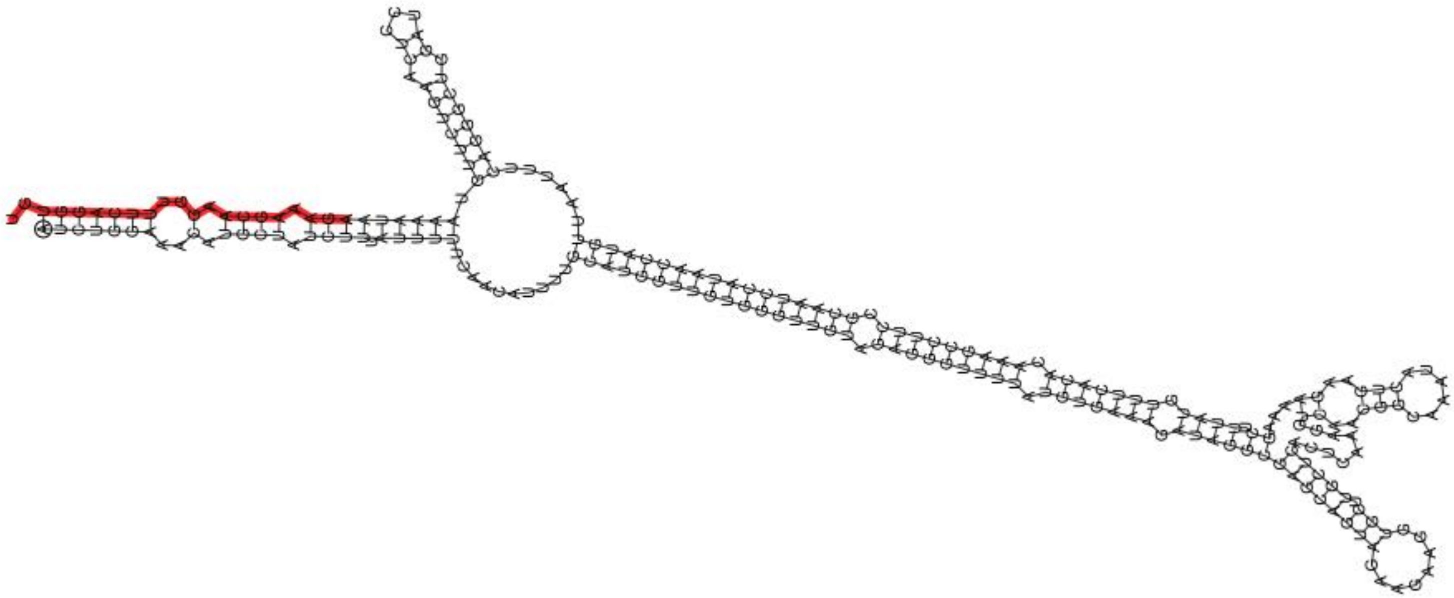

LC40

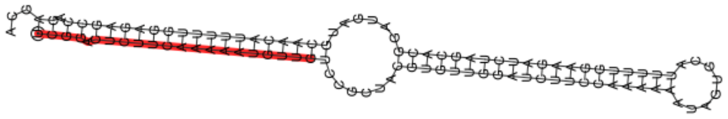

LC46

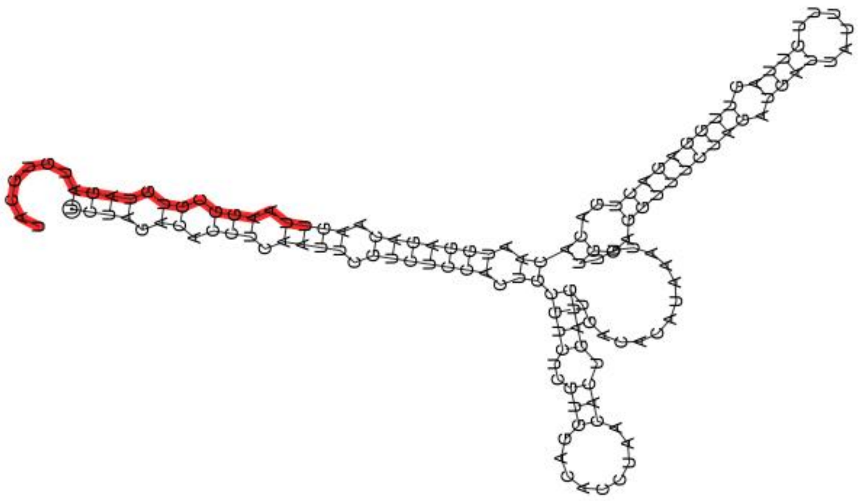

LC41
