# Supplementary material for: Identification of MicroRNAs and Target Genes in the Fruit and Shoot Tip of Lycium chinense: A Traditional Chinese Medicinal Plant
Source: PLoS One. 2015 Jan 14;10(1):e0116334. doi: 10.1371/journal.pone.0116334 (PMC4294688; doi:10.1371/journal.pone.0116334)
Supplement: S5 Table — (DOCX) [file pone.0116334.s007.docx]

**Table S5: The primer designed and candidate list for qRT-PCR**

| **Known miRNAs** | | | | | | | | | | | | |
| --- | --- | --- | --- | --- | --- | --- | --- | --- | --- | --- | --- | --- |
| **Sl. no** | **Name** | | | **Mature sequence** | | **SL (nt)** | | **SR** | **FR** | | **Fold /DE/SigL** | **Primer** |
| 1 | miR156a | | | UGACAGAAGAGAGUGAGCAC  (LA-UD)  TGACAGAAGAGAGTGAGCAC | | 20 | | 5 | 808 | | -7.48** | LCNt156a.F:  CGCCTGACAGAAGAGAGTGAGCAC |
| 2 | miR162 | | | UCGAUAAACCUCUGCAUCCAG  (RA-DU)  TCGATAAACCTCTGCATCCAG | | 21 | | 595 | 2801 | | -2.38** | LCSl162.F:  GCCCTCGATAAACCTCTGCATCCA |
| 3 | miR398 | | | UGUGUUCUCAGGUCGCCCCUG  (RA-DU)  TGTGTTCTCAGGTCGCCCCTG | | 21 | | 2 | 160 | | -6.46** | LCNt398.F:  TGTGTTCTCAGGTCGCCCCTG |
| 4 | miR166b | | | UCGGACCAGGCUUCAUUCCUC  (RA-DU)  TCGGACCAGGCTTCATTCCTC | | 21 | | 2587 | 5922 | | -1.34** | LCSt166b.F:  TCGGACCAGGCTTCATTCCTC |
| 5 | miR5301 | | | UGUGGGUGGGGUGGAAAGAUU  (LA-UD)  TGTGGGTGGGGTGGAAAGATT | | 21 | | 744 | 210 | | 1.69** | LCSl5301.F:  TGTGGGTGGGGTGGAAAGATT |
| 6 | miR394 | | | UUGGCAUUCUGUCCACCUCC  (LA-UD)  TTGGCATTCTGTCCACCTCC | | 20 | | 257 | 22 | | 3.41** | LCNt394.F  CGTTGGCATTCTGTCCACCTC |
| 7 | miR167 | | | UGAAGCUGCCAGCAUGAUCUA  (LA-UD)  TGAAGCTGCCAGCATGATCTA | | 21 | | 182 | 2563 | | -3.96** | LCSl167.F  CGGTGAAGCTGCCAGCATGATCTA |
| **Novel miRNAs** | | | | | | | | | | | | |
| 1 | | LC1 | UAGGGCGUUCGGAUCCUUCUGC  (LA-UD)  TAGGGCGTTCGGATCCTTCTGC | | 22 | | 2887 | | 18833 | -2.85** | | LC1.F:  TAGGGCGTTCGGATCCTTCTG |
| 2 | | LC17 | UCCAAUCUCCUCGCCCAUAUUU  (LA-UD)  TCCAATCTCCTCGCCCATATTT | | 22 | | 0 | | 95 | -12.33** | | LC17.F:  CGCTCCAATCTCCTCGCCCATA |
| 3 | | LC2 | UUGCCAAUUCCCCCCAUUCCGA  (RA-DU)  TTGCCAATTCCCCCCATTCCGA | | 22 | | 2662 | | 468 | 2.37** | | LC2.F:  TTGCCAATTCCCCCCATTC |
| 4 | | LC41 | UUAAGGCGUGUAGAUGUGCAU  (RA-DU)  TTAAGGCGTGTAGATGTGCAT | | 21 | | 245 | | 41 | 2.43** | | LC41.F:  CGGTTAAGGCGTGTAGATGTGCA |
| 5 | | LC4 | GAUCAUGUGGUAGCUUCACC  (RA-DU)  GATCATGTGGTAGCTTCACC | | 20 | | 85 | | 704 | -3.19** | | LC4.F:  CGGGATCATGTGGTAGCTTCACC |
| 6 | | LC32 | AUAAUACUUGGAAUAUGCCCU  (RA-DU)  ATAATACTTGGAATATGCCCT | | 21 | | 330 | | 127 | 1.24** | | LC32.F:  GGCGGGATAATACTTGGAATATGC |
| 7 | | LC14 | UAGAAAGAGUUUGUAGGCGAG  (RA-DU)  TAGAAAGAGTTTGTAGGCGAG | | 21 | | 56 | | 438 | -3.11** | | LC14.F:  GCGGTAGAAAGAGTTTGTAGGCGAG |

**Note:** SR=Shoot reads, FR=Fruit reads, Fold=Log2-fold-change, DE=Differentially expressed, Sigl=Significance level, RNA*= RNA::RNA* duplex, LA-UD= Left arm-Up to down in the 2ndary structure, LA-DU= Left arm- Down to Up in the 2ndary structure, RA-UD= Right arm-Up to down in the 2ndary structure, RA-DU= Right arm- Down to Up in the 2ndary structure.
