# Supplementary material for: Identification of MicroRNAs and Target Genes in the Fruit and Shoot Tip of Lycium chinense: A Traditional Chinese Medicinal Plant
Source: PLoS One. 2015 Jan 14;10(1):e0116334. doi: 10.1371/journal.pone.0116334 (PMC4294688; doi:10.1371/journal.pone.0116334)
Supplement: S7 Table — (DOCX) [file pone.0116334.s009.docx]

**Table S7: The designed primers for RLM 5’-RACE and targets annotation**

| **Target genes** | **Primer sequence (5’-3’)** | **Annotation** |
| --- | --- | --- |
| comp58287_c0: outer primer | ACATGCTGCAAGATTCCTTGGATAC | Squamosa promoter-binding-like protein 6 (SPL6) |
| comp58287_c0: inner primer | AACAGAAGAGCGACGGTTGACAC |  |
| comp45989_c0: outer primer | CATCAGCCCTTCACTGTCCACAT | Scarecrow-like protein 15 (SCL15) |
| comp45989_c0: inner primer | ACTGCTGTCTTCTCTCCTTCCATG |  |
| comp65633_c0: outer primer | TGCCAGGGTTCTTCAGAATCGTC | Auxin response factor (ARF) |
| comp65633_c0: inner primer | GCACACGGGTCATCATATCTAATC |  |
| comp32898_c0: outer primer | TCAACCGTCCTATTCCACAACATC | Glycine-rich RNA-binding protein (GRP2A) |
| comp32898_c0: inner primer | CAGCACCTAAGCAGGCTAGTCGTA |  |
